# Spatiotemporal Confinements of Distance-Dependent Emitters for Enhancing Plasmonic Signals

**DOI:** 10.1021/acsami.5c24749

**Published:** 2026-02-09

**Authors:** Yusuf Aslan, Esma Derin, Kutay Sagdic, Timuçin Emre Tabaru, Ali Karatutlu, Bülend Ortaç, Fatih Inci

**Affiliations:** † UNAM − National Nanotechnology Research Center, Bilkent University, 06800 Ankara, Turkey; ‡ Institute of Materials Science and Nanotechnology, Bilkent University, 06800 Ankara, Turkey; ∥ Sivas University of Science and Technology, Mecnun Otyakmaz Street No. 1, 58100 Sivas, Turkey

**Keywords:** Biosensor, surface plasmon resonance, metasurface, signal amplification, fluorescence, resonant-coupling

## Abstract

Surface plasmon resonance (SPR) is a common technique used for the real-time tracing of various analytes through refractive index–dependent resonance shifts. However, many plasmonic biosensors do not meet the clinical detection requirements for ultra-low concentration and low refractive index biomarkers. To address this challenge, researchers have explored unique labeling and interface modification strategies. One common strategy is utilizing fluorescence with plasmonic structures and enhancing the fluorescence intensity. However, these studies primarily focused on plasmon-enhanced fluorescence intensity, leaving the influence of fluorophores on reflection-/absorption-based plasmonic resonance shifts unexplored. Herein, we introduce a technique for amplifying the resonance shift of a plasmonic metasurface by confining the interdistance of fluorescence emitters. By adjusting nanospaces (∼4 to 20 nm), we couple surface plasmons with fluorescence in the near-field, achieving interdistance-dependent resonance shift behavior. This approach results in a 4.5-fold signal enhancement in the resonance shift for detecting conjugated proteins from complex matrices. In this regard, we utilize a plasmonic metasurface and distinct fluorescent emitters (FITC, Texas Red, streptavidin-quantum dot (QD) 525, and streptavidin-QD 625) with diverse excitation and emission assets. We also experimentally demonstrate a spectral blue shift of the plasmonic resonance through resonant coupling between QDs and surface plasmons, in contrast to the conventionally observed red shift. To hurdle the cost- and fabrication-related challenges in metasurfaces, we recycle off-the-shelf digital versatile discs (DVDs) into plasmonic metasurfaces due to their intrinsic nanograting structures, thereby significantly minimizing the cost down to $1.5. Moreover, we collect spatiotemporal signals using a palm-sized platform (5 cm × 10 cm x 1 cm) within 15 min that would be easily adapted into any settings possible. Consequently, this strategy paves the way for creating novel configurations and arrangements on a metasurface sensor to couple with fluorescence molecules while boosting the sensor’s analytical performance that would be potentially integrated with biosensing applications in disease diagnostics.

## Graphical abstract

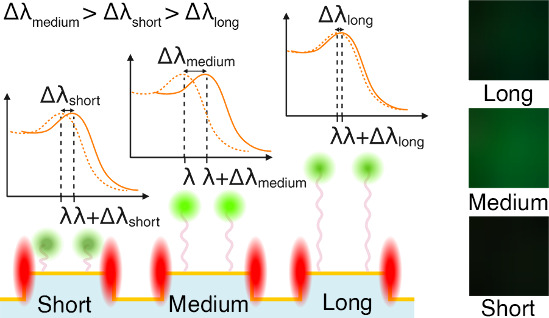

## 1 Introduction

Emerging analytical micro- and nanoscale technologies have prevailed in comprehensive applications in sensor technologies to recognize biological compounds, such as membrane interactions with biomolecules,^1^ antigen–antibody recognition,^2^ DNA sensing,^3^ and protein–protein or protein–surface interactions.^4,5^ Recently, the range and variety of detected biomarkers have been largely increased thanks to plasmonic biosensors.^6–9^ These platforms enable label-free, rapid, and real-time detection of clinically relevant targets such as extracellular vesicles,^10^ circulating DNA^11,12^ and RNA fragments,^13^ cardiac troponins,^14^ and C-reactive proteins.^15^ This capability arises from monitoring changes in the local refractive index near the plasmonic sensor interface, where the immobilization of adsorbates on the plasmonic surface increases the local refractive index and causes a red shift in the plasmonic resonance wavelength.^16^ This fundamental principle allows real-time monitoring of affinity binding, target–ligand kinetics, and quantitative molecular detection.^17^

Commercial SPR instruments are widely used for designing immunosensors for drug discovery,^18^ recognition-element (antibody, molecularly imprinted polymers (MIPs), and aptamers) characterization,^19–21^ diagnostics,^22^ and so on. However, they have notable drawbacks that hinder their large-scale usage in resource-limited settings and high-throughput screening. The main drawbacks are bulky and non-portable configurations, limited sensitivity, and the requirement for phase-matching optical elements, thereby increasing the overall cost of the equipment.^23^ Plasmonic metasurfaces offer an attractive alternative over the conventional SPR biosensors by improving sensitivity and eliminating the cost linked with the phase-matching required optical element.^24^ We have previously demonstrated the conversion of DVDs into plasmonic metasurfaces and successfully sustained Fano-resonance for biotarget tracing at a low-cost and portable setup, suited for POC applications.^25^ We further demonstrated the detection of micro- and nanoplastics over the metasurface by plasmon enhanced fluorescence (PEF).^26^ Though, the usage of pretemplated surfaces limits the sensitivity of the metasurface up to a certain level determined by the dimensions of the grooves. Limited sensitivity remains a common challenge in plasmonic biosensors, as many biomarkers require more responsive platforms due to their minute concentrations in biological fluids^27^ and inherently low refractive index contrast with the surrounding medium.^28^ To overcome these challenges, various signal amplification strategies have been developed on target labeling and/or interface modifications. These strategies mostly rely on active^29^ or passive^30^ contribution of nanostructures, including metallic (gold, silver, or bimetallic) nanoparticles,^31^ nanoislands,^10^ fluorophores,^32^ quantum dots,^33^ and two-dimensional materials (graphene and more).^34^ Among these signal amplification methods, fluorescence has been extensively utilized with plasmonic structures for enabling PEF and tracking biomarkers with fluorescence intensity.^35^ These platforms require optimization of several performance parameters for achieving maximum fluorescence enhancement. The main contributors are the interdistance and spectral overlap between the fluorophore and the plasmonic structure along with the side contributors. The interdistance have been previously controlled by tuning the molecular weight of polymeric spacers (such as PEG),^36–38^ length of DNA,^39^ and size of polymer nanobeads.^40^ Among these strategies, hydrophilic PEG polymers provide stable configurations for adjusting the interdistance in aqueous solutions.^37^ Once these parameters are optimized, PEF biosensors utilize the fluorescence intensity for the detection of biomarkers. However, the presence of fluorophore can also induce a change in reflection/absorption based plasmonic resonance by means of a spectral shift. Such an example was earlier reported by Nguyen Thi et al., where they surprisingly observed non-oscillatory behaving QD-induced blue shifts over optical fiber-based surface plasmons.^41^ This outcome highlights the necessity of examining the plasmonic resonance shifts under the presence of fluorophores with different interdistance and spectral overlap conditions.

In this study, we systematically tuned the interdistance and spectral overlap between fluorophores and the plasmonic metasurface to evaluate the changes in plasmonic resonance. Plasmonic metasurfaces were fabricated over the DVD surface by employing multiple metallic layers (Ti, Ag, and Au). Finite-difference time domain (FDTD) analysis was employed to analyze electric field strength and distribution at the groove structure of the plasmonic metasurface. Additional FDTD analysis was performed to study the impact of the fluorophore (dipole) over the plasmonic metasurface with varied interdistance. The interdistance between fluorophores and plasmonic metasurface were determined with three different surface modifications (Figure 1): short 3-Mercaptopropanyl-*N*-hydroxy-succinamide ester (3-MNHS), medium (SH-PEG 600-Biotin), and long (SH-PEG 2000-Biotin). The impact of fluorescence on the absorbed plasmonic resonance shift was analyzed by separately immobilizing avidin and fluorophore conjugated avidin proteins (avidin-FITC) for each surface modification. The impact of the spectral overlap was characterized by tuning the excitation/emission assets of the fluorophore conjugated avidin proteins (avidin-FITC, avidin-Texas Red, streptavidin QD 525, and streptavidin QD 625) on the medium-distance surface modification. We also observed QD-induced blue shift on medium-distance modification, consistent with previous reports of Thi et al.^41^ Moreover, PEF of a conjugated protein (avidin-FITC) was additionally recorded for each surface modifications. Lastly, the performance of the optimized interdistance and spectral overlap configuration was assessed in artificial urine. Therefore, this strategy offers a notable potential to improve sensitivity up to 4.5 times; shorten turnaround time (15 min); reduce related costs down to $1.5; and enable portability as a palm-size device; thereby broadening its expansion to multiple settings.

**Figure 1:**
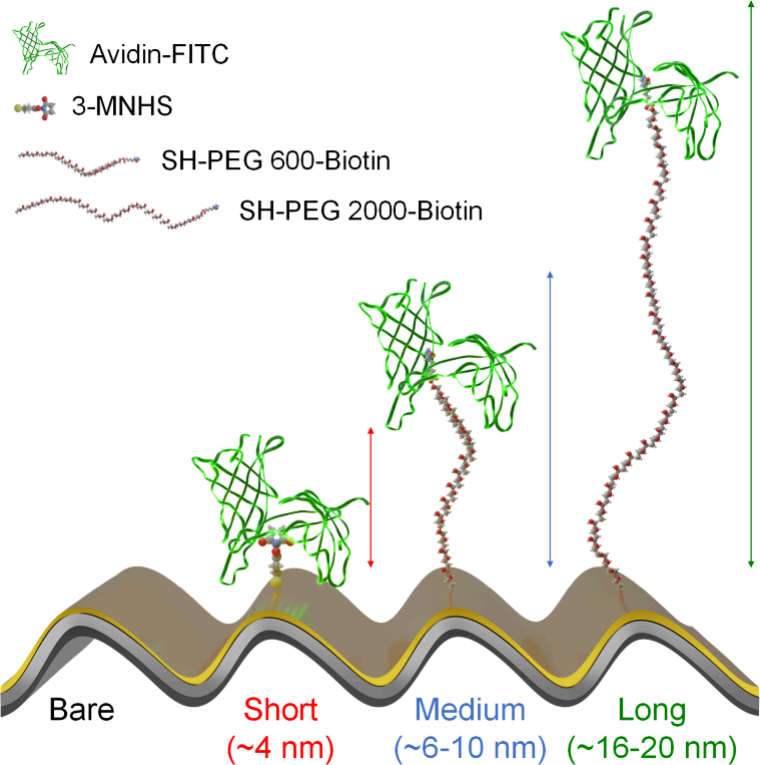
Schematic illustration of distance-controlled surface modifications on the plasmonic metasurface. 3-MNHS is employed for short-distance functionalization, yielding an estimated ∼4 nm separation between avidin–FITC and the plasmonic metasurface. Likewise, SH-PEG600-biotin is used for medium-distance modification, resulting in an approximate separation of 6–10 nm. SH-PEG2000-biotin enables long-distance modification, providing an estimated separation of 16–20 nm.

## 2 Results and Discussion

### 2.1 Fabrication of the Plasmonic Metasurface

The plasmonic metasurface was fabricated by thermal evaporation of metallic layers above a wet etched DVD substrate. The DVD substrate contains nanoscale grooves in a polycarbonate (PC) template, and these intrinsic nanostructures eliminate the need for costly lithography techniques. The wet-etching step allows the adjustment of the width of grooves, while deposited metallic layers enable the excitation of multiple optical modes and plasmonic resonance. The fabrication of the metasurface involves sample preparation, wet etching, and physical vapor deposition of metallic layers. All steps were carried out the following established protocols.^25,42^

### 2.2 Topography Characterization of DVD and Metasurface

The wet etching process primarily affects the grating structure by altering its height and width, while keeping the periodicity constant. The etching duration (60s) was determined on our previous study considering the optical response of the metasurfaces,^25^ while we only demonstrated the corresponding morphological changes in this study. The DVD was wet etched for 60 s, and the effects of etching duration (10, 20, 30, 60, 90, and 120 s) on morphological changes (Figure S1) of the fabricated plasmonic metasurfaces were presented in the **Supporting Information**. The groove dimensions (height, period, and width) of the wet-etched DVD substrate and plasmonic metasurface were characterized through Scanning Electron Microscopy (SEM) and Atomic Force Microscopy (AFM) (Figure 2a).

**Figure 2:**
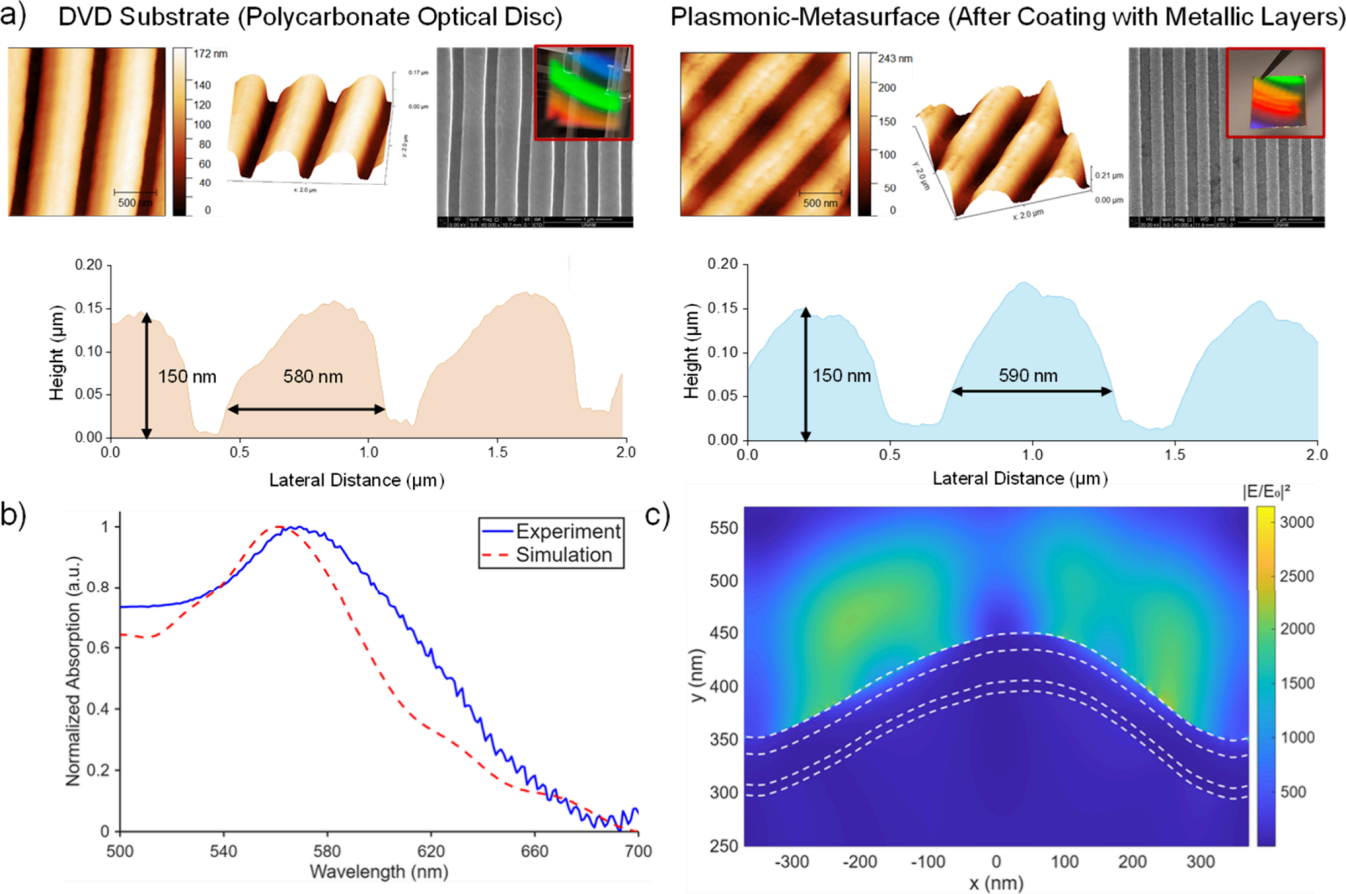
Surface topography and optical characterization of the plasmonic metasurface. a) SEM and AFM images of the metasurface, together with the corresponding line profiles. b) Normalized experimental (red dashed line) and simulated (blue solid line) absorbance spectra. c) Electric field distribution of the plasmonic metasurface at the resonance wavelength.

The wet-etched PC-templated DVD exhibited smooth surfaces and edges with a root-mean-square roughness (Rq) of 1.9 nm (Figure S2a), while the plasmonic metasurface has a slightly rougher surface with a Rq of 5.3 nm (Figure S2b). The wet-etched DVD exhibited groove height and width values of approximately 150 and 580 nm, respectively (Figure 2 **a, left**). After metal deposition, the metasurface preserved the underlying geometry, showing similar dimensions of ∼ 150 nm in height and ∼ 590 nm in width (Figure 2 **a, right**). The periodicity of the grooves remained at ∼ 740 nm for both samples.

The cross-sectional characterization of the plasmonic metasurface was performed by Focused Ion Beam (FIB)-SEM (Figure S3). The plasmonic metasurface was ion-milled in a controlled manner, and the internal structure of the deposited metallic layers was exposed (Figure S3a). The cross-section showed that the total thickness of the metallic layers (Ti–Ag–Au) deposited on the DVD substrate was approximately 63.5 nm (Figure S3b-c). The intended deposition thickness was 55 nm in total (10 nm Ti, 30 nm Ag, and 15 nm Au), corresponding to an error of ∼ 15%. The metasurface cross-section demonstrated a conformal metal coating and preserved the groove morphology of the substrate.

### 2.3 Numerically Calculated E-field and Resonance of the Plasmonic Metasurface

Once the sensor dimensions were reached, we further evaluated the electromagnetic behavior of the metasurfaces through numerical FDTD simulations. To achieve this, the three-dimensional (3D) AFM profile of bare PC-templated DVD was imported into a simulation environment as the base geometry, and the subsequent metallic layers (10 nm Ti, 30 nm Ag, and 15 nm Au) were placed above the base layer. By this, we obtained a realistic scenario of our metasurface electromagnetic response. The metallic layers were chosen in a strategic manner. Ti provided a strong adhesion layer for Ag and Au layers. Ag is one of most utilized noble metals for low optical loss and high field enhancement, well suited for plasmonics. Au was primarily chosen for its chemical stability and biocompatibility.^44^ The thickness values of the metallic layers were optimized in a previous study to minimize optical loss and increase field enhancement.^25^ The computational model of the plasmonic metasurface predicts a plasmonic resonance at 561 nm, which agrees well with the experimentally observed resonance at 569 nm (Figure 2b). The slight variation in resonance peak positions and the full width at half-maximum (fwhm) between the experimental and simulated spectra is primarily attributed to the surface roughness of the evaporated metal layers. In addition, the circular pattern of the optical disc and batch-to-batch variations among commercial DVD contribute to minor deviations in the resonance position and absorption characteristics. The simulated electric field intensity is concentrated along the slope of the grating (Figure 2c).

The influence of FITC emitters on the absorption spectra of the metasurface was numerically analyzed by simulating a single dipole (emission wavelength: 516 nm) placed at the center of the grating (Figure 3i). The interdistance between the metasurface and dipole was varied from 2 to 20 nm. The intensity of the absorption spectra changed with varying interdistance. This result demonstrated a distance-dependent coupling between the dipole and the plasmonic surface (Figure 3j).

**Figure 3:**
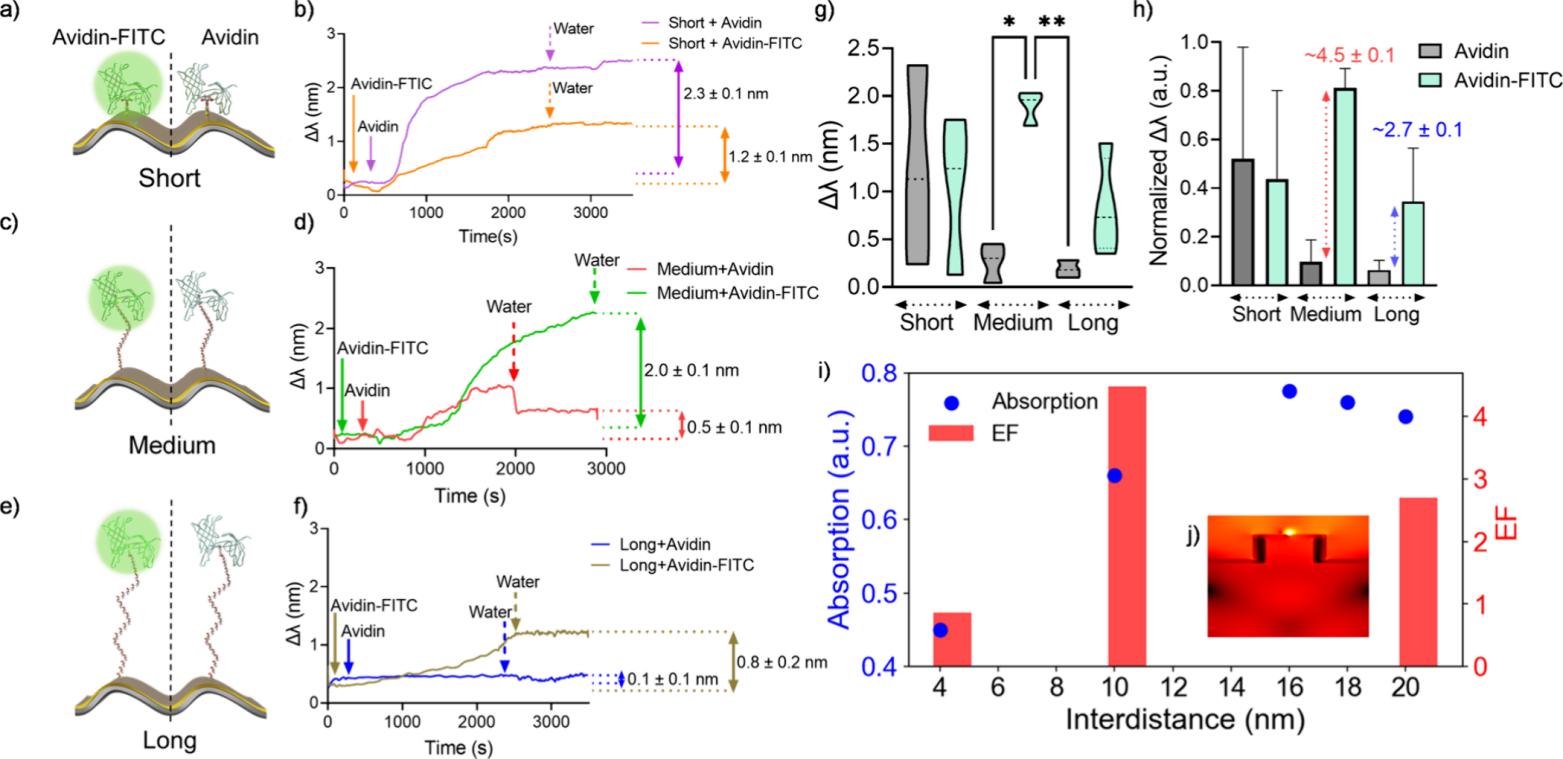
Evaluation of avidin–FITC and avidin binding on distance-controlled surface modifications via resonance wavelength shift (Δλ). Avidin–FITC (left) and avidin (right) were separately immobilized, and Δλ values (mean ± standard deviation, *n* = 3) were measured for (a,b) short-, (c,d) medium-, and (e,f) long-distance modifications. g) Summary of Δλ values and corresponding enhancement factors (EFs) presented as violin plots. h) Normalized Δλ data (scaled from 0 to 1) shown as bar plots. i) Simulated absorption maxima and calculated enhancement factors demonstrating correlation with experimental results. Inset (j) shows the electric field distribution of a dipole emitter (516 nm) on the plasmonic metasurface. Statistical analysis was performed using a nonparametric Kruskal–Wallis test; significance is indicated as *p < 0.05 and **p < 0.01.

### 2.4 Benchmarking the Performance Parameters of Plasmonic Metasurface

The performance parameters of a plasmonic metasurface typically evaluated by refractive index unit (RIU) sensitivity (S) and the resonance shift upon a change in the RIU of the surrounding medium, which is also known as figure of merit (FoM).^45^ Once the plasmonic metasurface was fabricated, the RI sensitivity was investigated in our optical setup (Figure S4) by systematically varying the RI of the surrounding medium. For this, various glycerol concentrations (1–70%) were applied to the surrounding medium, and the corresponding absorption spectra were recorded (Figure S5). We initially applied water on the plasmonic metasurface as a background and observed two different resonances: (i) a dip at 533.24 nm and (ii) a peak at 567.38 nm, and these resonances red-shifted proportionally with increasing glycerol concentrations (Figure S5a). The peak resonance was more sensitive to the refractive index changes, and we hence continued to monitor resonance peak (ii) throughout this study. The end and real-time measurements were plotted through our in-house MATLAB GUI interface (Figure S5a-b). The real-time measurements were reported by applying adjacent averaging to the raw data, which reduced the resolution of the standard deviation (Figure S6). Overall, we observed linear increments in the resonance peak in response to RI alterations induced by glycerol injections (Figure S5c).

The S value was calculated through division of the resonance shift by the corresponding RI change (eq 1), which is 383.25 nm/RIU. The FoM values were calculated through division of the S values for each resonance shift by the corresponding fwhm values (eq 2), and all the data were provided in Table S1. The FOM values varied between ∼ 12.5 and 16, and the FoM values increased as the glycerol concentration increased. Higher values of FOM indicate better sensor performance, therefore it is predicted that the sensor would potentially be able to achieve the detection in matrices with a high RI, such as body fluids (urine, serum, blood, and plasma).^46–50^

$$\mathrm{Refractive}\;\mathrm{Index}\;\mathrm{Sensitivity}=\frac{\Delta \lambda }{\Delta n}$$ 1

$$\mathrm{Figure}\;\mathrm{of}\;\mathrm{Merit}=\frac{\mathrm{Refractive}\;\mathrm{Index}\;\mathrm{Sensitivity}}{\mathrm{FWHM}}$$ 2

### 2.5 Numerical Flow Simulations of the Microfluidic Chip

The surface modifications were applied after the microfluidic was integrated onto the metasurface. The microfluidic chip and operating flow rate were specifically designed to maximize the loading capacity of surface modifications and the capture efficiency of the proteins. For this, the shear stress inside the microfluidic chip was numerically calculated at different fluid flow rates by using the finite element method (FEM). Inspired by our previous work,^51^ we designed a hexagonal-shaped microfluidic chip and numerically modeled the velocity profiles at various flow rates, as well as the temporal shear stress at different microchannel heights (Figure S7). Using the analytical model, the microfluidic channel height, fluid flow rate, and adhesion time were optimized for the surface modification loading capacity and protein capture efficiency. Details of the analytical model optimization are provided in the Supporting Information **.**

### 2.6 Surface Modifications

Three different surface modifications (short, medium, and long) were used to tune the distance between the plasmonic metasurface and fluorophore-conjugated proteins. The interdistance values for each surface modification were estimated rather than directly measured. Some studies estimate the spacer length using dynamic light scattering (DLS), which measures changes in hydrodynamic size before and after PEG attachment. However, this method works only for plasmonic nanoparticles and is not applicable to our metasurface. In our case, the corrugated nature of the DVD-templated plasmonic metasurface prevents direct measurement of the thickness of surface modifications using common techniques such as AFM, ellipsometry, or DLS. Therefore, interdistance values were estimated using literature-reported molecular dimensions and established calculations. The size of avidin and streptavidin was also incorporated into these estimations.

The short-distance modification was enabled through 3-Mercaptopropanyl-*N*-hydroxy-succinamide ester (3-MNHS). This chemical linker has a molecular size less than 1 nm, so the effective interdistance was primarily determined by the dimensions of the avidin- or fluorophore-conjugated avidin/streptavidin protein. The size of avidin or streptavidin was reported as ∼ 4 nm.^52^ Thus, the interdistance for the short-distance modification was estimated to be ∼ 4 nm.

For the medium- and long-distance modifications, PEG polymers were preferred since they were extensively studied in the literature due to their hydrophilicity, biocompatibility, and predictable contour-length scaling with adjustable molecular weight. Prior studies commonly utilized thiol-terminated PEG to anchor onto gold nanoparticles with the opposite end functionalized (often with amine groups) to attach a specific fluorophore.^36–38^ Such designs allow fine-tuning of the interdistance for a single application-specific fluorophore but do not readily accommodate multiple fluorophores. In contrast, we employed biotinylated PEG polymers (SH-PEG-Biotin) that enable application-independent fluorophore coupling through the universal avidin–biotin interaction. This approach provides a modular platform compatible with a wide range of fluorophore-tagged proteins without altering the underlying PEG spacer chemistry.

The interdistances of the medium- (SH-PEG 600-biotin) and long-distance modifications (SH-PEG 2000-biotin), were calculated based on the number of repetitive ethylene glycol molecules (n) found in these polymers. The molar mass of PEG was calculated by the following formula, (MW_PEG_ = 18.02 + (44.05*n*) g/mol).^53^ This corresponds to approximately 13 and 45 subunit (ethylene glycol) for PEG 600 and PEG 2000, respectively. Each ethylene glycol unit contributes 0.278–0.358 nm to the contour length, depending on covalent-bond orientation.^54^ PEG polymer forms a flexible random coil in aqueous solutions, which can adopt various orientations, such as tilted, partially extended, or coiled. These orientations depend on numerous factors, including interface geometry, packing density, and solvent properties.^55–57^ The contour length of PEG provides a well-accepted upper-bound estimate of the end-to-end distance of surface-tethered PEG chains. Similar contour-length approaches are widely used for PEGylated surfaces and polymer-spacer systems in other studies.^37,54,58^ Additionally, the avidin–biotin complex was reported between 4.2 and 5.8 nm.^59^ Considering the length of PEG polymers and avidin–biotin complex, the medium- and long-distance modifications are estimated to provide interdistances of approximately 7.9–10.5 and 16.7–21.9 nm, respectively.

### 2.7 Surface Modification Characterization by XPS

The presence of elemental components of each surface modification were assessed and verified by X-ray photoelectron spectroscopy (XPS, K-Alpha, ThermoFisher Scientific, USA). XPS analysis acquired C 1s, O 1s, N 1s, S 2p, and Au 4f spectra, as well as their atomic ratio (Figure S8). The detailed findings of the XPS analysis were provided in Supporting Information **.**

### 2.8 Signal Amplification by Tuning the Interdistance via Surface Modifications

The impact of the fluorophore on the plasmonic resonance shift (Δλ) was evaluated for each surface modification. Avidin was first used as the nonfluorescent control group, and Δλ was recorded on each surface modification. Then, avidin-FITC was introduced separately as the fluorophore-conjugated protein, and its effect on Δλ was analyzed under identical conditions. Avidin-FITC was selected for optimizing the interdistance because its emission showed the highest overlap with the plasmonic resonance (except for QD 525) (Figure S9). QDs were analyzed separately, as the coupling between QDs and plasmonic resonance has been previously reported to induce abnormal blue shift.^41^

We began the interdistance tuning process by introducing avidin on the short-distance modification, which led to a Δλ of 2.32 ± 0.1 nm (Figure 3 **a-b**). In comparison, addition of avidin-FITC on the short-distance modification reduced the Δλ to 1.24 ± 0.1 nm, indicating that the presence of FITC lowered the Δλ value (Figure 3 **a-b**). This Δλ reduction on short-distance modification corresponded to a 15% signal loss compared with the avidin (Figure 3 **g-h**). This may be attributed to the non-radiative energy transfer between the FITC emitters and the surface plasmons, which reduced the RI sensitivity in the presence of FITC.

For the medium-distance modification, avidin induced a Δλ of 0.45 ± 0.1 nm, whereas the avidin-FITC demonstrated a Δλ of 2.03 ± 0.1 nm (Figure 3 **c-d**). This result indicated an ∼ 4.5-fold signal enhancement in the presence of FITC compared to the control group (Figure 3 **g-h**). The strong enhancement suggests more efficient plasmon–fluorophore coupling at this intermediate distance, where radiative processes dominate over nonradiative quenching.

For the long-distance modification, no significant Δλ (0.1 ± 0.1 nm) was observed for avidin adhesion, whereas the Δλ for avidin-FITC yielded 0.88 ± 0.2 nm (Figure 3 **e-f**). Once again, the presence of FITC on the long-distance modification led to an increase in Δλ compared to that in the control group, resulting in an ∼ 2.7-fold signal enhancement (Figure 3 **g-h**). At this longer distance, the fluorophore moves further out of the near-field range, and consequently, the efficiency of plasmonic coupling may diminished. Overall, the highest signal amplification was recorded on the medium-distance modification, suggesting the effective coupling between plasmonic resonance and fluorophore emission was achieved at 7.9–10.5 nm.

We further simulated a dipole over the plasmonic metasurface via FDTD (Figure 3j). The dipole was emitting a single wavelength (516 nm), which mimicked the emission of FITC, and it was positioned right above the metasurface with an initial 4 nm interdistance. The interdistance was gradually increased from 4 to 16 nm, and the numerically calculated absorption spectrum was recorded separately. The absorption maximum of the recorded spectra increased on the medium-distance modification and reduced on long-distance modification, demonstrating a trend similar to that of experimentally realized enhancement factor (signal amplification).

### 2.9 Signal Amplification by Tuning the Spectral Overlap Between Plasmonic Resonance and Fluorophore Emission

The most effective interdistance for signal amplification was the medium-distance modification, corresponding to approximately 7.9–10.5 nm. Following this optimization, we aimed to further tune the spectral overlap between the plasmonic resonance and different fluorophore emission profiles over medium-distance modification. For this, we separately introduced avidin-Texas Red, streptavidin-QD 525, and streptavidin-QD 625 onto the medium-distance modification and compared their Δλ responses to those obtained from avidin and avidin-FITC.

Initially, we introduced avidin-Texas Red, which demonstrated an Δλ of 0.57 ± 0.4 nm (Figure 4 **a-b**). This Δλ was ∼ 2.1 times greater than the Δλ obtained for avidin alone (Figure 4 **c-d**). However, the Δλ generated by avidin-FITC remained 2.5 times larger than that of avidin–Texas Red (Figure 4 **c-d**).

**Figure 4:**
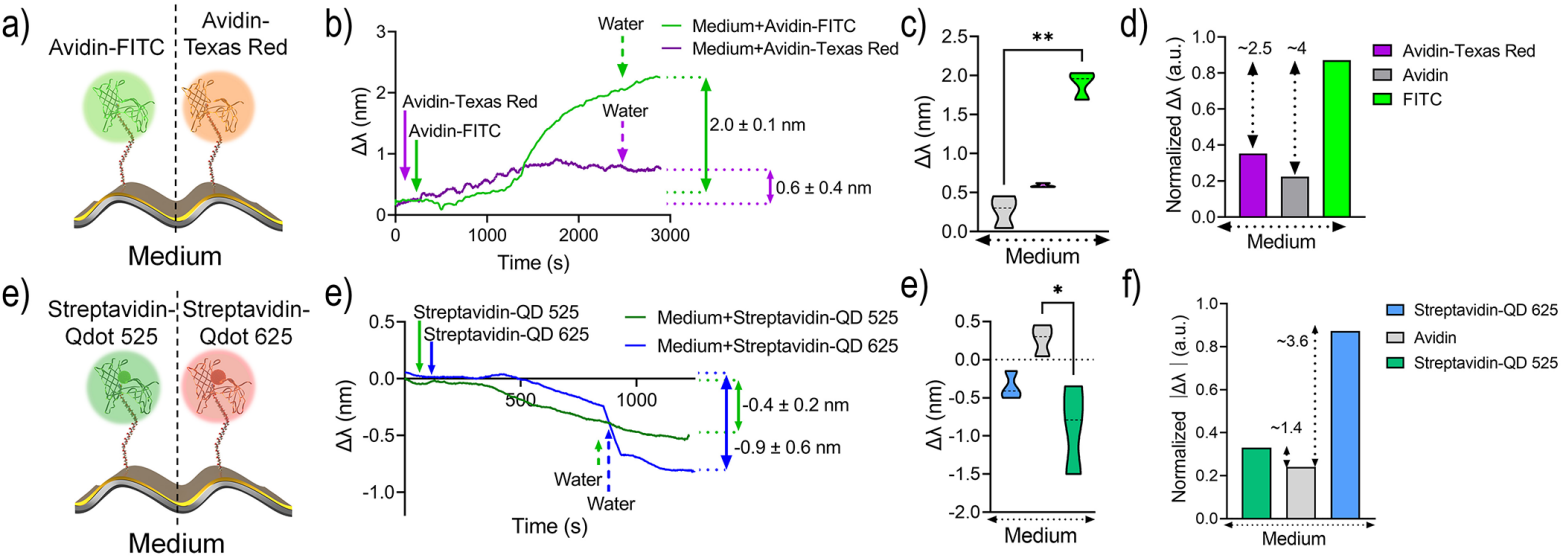
Resonance wavelength shifts (Δλ) of the plasmonic metasurface following the binding of avidin–FITC, avidin–Texas Red, streptavidin–QD525, and streptavidin–QD625 on the medium-distance surface modification. a) Binding of avidin–FITC (green, left) and avidin–Texas Red (orange, right). b) Corresponding Δλ values for avidin–FITC (green) and avidin–Texas Red (purple). c) Violin plots and d) normalized (0–1) bar plots summarizing Δλ distributions for avidin (gray), avidin–FITC (green), and avidin–Texas Red (purple). e) Binding of streptavidin–QD525 (green, left) and streptavidin–QD625 (red, right). f) Corresponding Δλ values for streptavidin–QD525 (green) and streptavidin–QD625 (blue). g) Violin plots and h) normalized (0–1) bar plots summarizing Δλ distributions for avidin (gray), streptavidin–QD525 (green), and streptavidin–QD625 (blue). Statistical analysis was performed using a nonparametric Kruskal–Wallis test; significance is indicated as * *p* < 0.05 and ** *p* < 0.01.

Next, streptavidin-QD 525 and streptavidin-QD 625 produced Δλ values of −0.9 ± 0.6 and −0.4 ± 0.2 nm, respectively (Figure 4 **e-f**). Unlike other fluorophores, both QD conjugates (streptavidin- QD 525 and streptavidin-QD 625) exhibited a blue shift. A previous study also reported a QD-induced blue shift on fiber surface plasmons.^41^ The study attributed this abnormal negative RI change to the gain characteristics of excited QDs and their long-lived excited state.

After normalizing the absolute Δλ values, streptavidin–QD 525 exhibited approximately a 3.6-fold signal enhancement, whereas streptavidin–QD 625 showed only a 1.4-fold increase relative to the control (Figure 4 **g–h**). A common feature between avidin–FITC and streptavidin–QD 525 is their substantially larger spectral overlap with the plasmonic resonance compared to avidin–Texas Red and streptavidin–QD625 (Figure S9).

The Δλ trends observed across different fluorophores suggests that the near-field plasmonic coupling efficiency is strongly dispersive and influenced by the degree of spectral overlap, with greater overlap leading to stronger coupling and larger Δλ values.^60^ In addition, fluorophores other than FITC exhibited large standard deviations in Δλ, which indicated a high variability in their observed plasmonic response. Therefore, green-emitting fluorophore-plasmon pairs (avidin-FITC and streptavidin-QD 525) demonstrated significantly greater signal enhancement compared to red-emitting fluorophore-plasmon pairs (avidin-Texas Red and streptavidin-QD 625) (Figure S10).

### 2.10 Visualizing the Fluorescence-Plasmon Coupling

The intensity of PEF depends on the structure of the plasmonic surface, the interdistance between fluorophore and plasmonic surface, and the spectral overlap between plasmonic absorption and excitation and emission spectra of the fluorophore. Initially, the immobilization of avidin-FITC was visualized on the medium distance modification (Figure S11a). To achieve this, we set three different conditions: (i) medium-distance modification, (ii) the surface under flow of avidin-FITC, and (iii) the surface after PBS washing to remove unbound fluorophores. In first condition (i), the medium-distance modification exhibited only background noise, and no fluorescence was observed. This resulted in low intensity mean gray value with high count frequency in the histogram plot (Figure S11b). On condition (ii), avidin-FITC was introduced, and freely diffusing fluorophores increased the background intensity. Additionally, discrete green fluorescent spots appeared, corresponding to immobilized avidin-FITC molecules. This led to a higher mean gray value and broader intensity distribution in the histogram. In the last condition (iii), PBS washed unbound avidin-FITC molecules, and only the immobilized fluorophores remained. Consequently, the background intensity decreased, and the histogram shifted to intermediate intensity between the (i) and (ii) conditions. The normalized mean gray values confirmed this trend (Figure S11c).

Following the same strategy, we visualized short-, medium-, and long-distance modifications (Figure 5 **a-d**). Short-distance modification demonstrated the lowest fluorescence intensity, indicating a quenching effect compared with both medium- and long-distance modifications (Figure 5 **a, b, and c**). The medium-distance modification outperformed the short- and long-distance modifications in fluorescence intensity, with ∼ 3.7-fold and ∼ 2.6-fold increases, respectively (Figure 5a). On the long-distance modification, the fluorescence intensity was slightly higher than that on the short-distance modification but remained significantly lower than that on the medium-distance modification (Figure 5 **a, b, and c)**. These results align with the signal enhancements obtained from the Δλ values (Figure 3 **g-h**). The expected nonradiative energy transfer influencing the Δλ result of short-distance modification was supported by the observed quenching in fluorescence intensity. For both medium- and long-distance modifications, we anticipated an enhancement in the radiative coupling, which corresponded with the fluorescence enhancement, peaking at the medium-distance modification.

**Figure 5:**
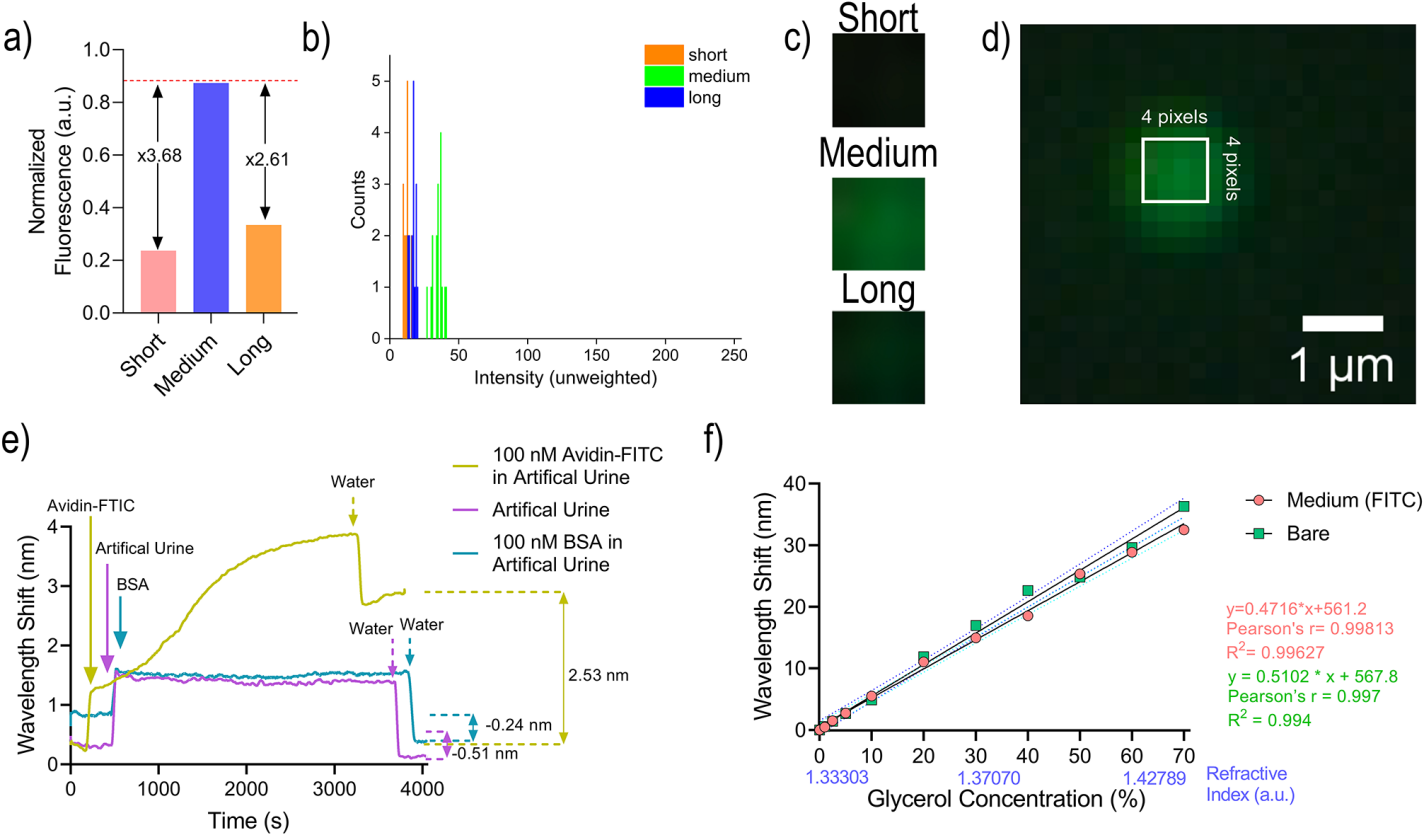
Fluorescence intensity and plasmonic sensing performance of distance-modified metasurfaces in artificial urine. a) Normalized fluorescence intensity, b) fluorescence intensity histogram, and c) regions of interests (ROIs) of avidin–FITC on short-, medium-, and long-distance surface modifications. d) Wide-field fluorescence image of avidin–FITC on the medium-distance modification. e) Resonance wavelength shift (Δλ) of the plasmonic metasurface following separate additions of avidin–FITC and BSA on the medium-distance modification in artificial urine. After avidin–FITC binding, refractive index sensitivity analysis was performed. f) Δλ response of the avidin–FITC–functionalized medium-distance modification and the bare metasurface in glycerol solutions (1–70%).

### 2.11 Detecting Biomolecules in Complex Matrices

Until this stage, fluorescence emitter-plasmon interactions were analyzed in simple aqueous environments (ddH_2_O and PBS). However, most biological fluids have significantly more complex compositions. To approximate a biologically relevant sample, we utilized artificial urine to evaluate the sensor performance by monitoring the Δλ response. The medium-distance modification was first monitored in PBS, and then artificial urine spiked with either avidin-FITC (i) or BSA (ii) was introduced into medium-distance modification (Figure 5e). Artificial urine alone was also assessed as a reference. To determine the Δλ values, a water wash was applied to remove any unbound materials.

Following exposure to avidin-FITC, a Δλ of 2.53 nm was observed, whereas BSA produced a Δλ of −0.24 nm (Figure 5e), suggesting that the fluorescence emitter-plasmon pair provides a stable and distinct signal in an artificial urine matrix. In the case of BSA, a minimal Δλ was observed, indicating little or no non-specific binding between BSA and the medium-distance modification. After the introduction of artificial urine, the subsequent water wash led to a blue shift of −0.51 nm, attributing to changes in the refractive index of the medium (Figure 5e).

To further assess the refractive index sensitivity of the avidin-FITC-coupled metasurface, various glycerol solutions (1%–70%) were applied over the medium-distance modification (Figure 5f). Using eq 1, the refractive index sensitivity of the avidin-FITC-coupled metasurface was calculated as 343.5 nm/RIU (Figure 5f), slightly lower than that of the bare metasurface (383.25 nm/RIU) (Figure S5c **)**. Additionally, we calculated fwhm and FOM values for the avidin-FITC-coupled metasurface (1%–70%) on the avidin-FITC-coupled metasurface (Table S2). Compared with the bare metasurface (Table S1), the avidin-FITC-coupled metasurface demonstrated the reduced fwhm values and enhanced FOM values. This result indicated improved sensitivity relative to the bare metasurface.

## 3 Conclusion

In this study, we demonstrated signal amplification of a DVD-templated plasmonic metasurface by using distance-dependent fluorophore-plasmon coupling. The interdistance between fluorophores and the plasmonic metasurface was finely tuned using three different surface modifications. These surface modifications were either chemical linkers or biotinylated PEG polymers, spanning approximately 4–20 nm. The molecular weight of the PEG polymers allowed precise adjustment of this interdistance, while the biotin enabled the versatile attachment of fluorophore-conjugated avidin or streptavidin.

Among the three configurations, the medium-distance modification (∼7.9–10.5 nm) produced the highest signal amplification due to the effective coupling between the fluorophore and surface plasmons. Further, different fluorescent dyes (avidin-FITC, avidin-Texas Red, streptavidin-QD 525, and streptavidin-QD 625) were examined for medium-distance surface modification. Among these, avidin-FITC generated the highest signal amplification (∼4.5-fold) over the medium-distance modification, owing to its strong spectral overlap with the plasmonic resonance. Further, both streptavidin-QD 525 and streptavidin-QD 625 demonstrated a QD-induced blue shift, an abnormal negative RI change due to the gain characteristics of long-lived excited states of QDs. Additionally, plasmon-enhanced fluorescence was observed for avidin-FITC with medium-distance modification, confirming efficient radiative coupling in this regime. Lastly, plasmon-enhanced fluorescence was observed for avidin-FITC on the medium-distance modification, confirming efficient radiative coupling in this regime.

This platform demonstrates the effective detection of avidin and avidin/streptavidin fluorophore conjugates and potentially proposes the implementation of biotinylated recognition elements (antibodies, enzymes, nucleic acids, aptamers, and MIPs) for highly sensitive biomarker detection. Our metasurface was fabricated using a lithography-free approach, which minimizes clean-room usage and reduces the fabrication cost. Additionally, our optical measurement setup is portable and facilitates its potential clinical and enviromental applications in low-resource or remote settings. Apart from POC applications, our interdistance-dependent surface modification strategy could be adapted for the optical mapping of sulfur vacancies in two-dimensional transition metal dichalcogenides, such as MoS_2_,^61^ by controlling the Förster resonance energy transfer between the fluorescence emitter and surface defects.

## 4 Experimental Section

### 4.1 Plasmonic Metasurface Fabrication

The plasmonic metasurface was fabricated by thermal evaporation of metallic layers above the wet etched DVD substrate. For this, 10 nm of Ti was coated over the DVD as an adhesive layer for plasmonic noble metals. Then, Ag (30 nm) and Au (15 nm) were coated over the Ti layer. Detailed information about wet etching and evaporation process were shown in the Supporting Information **.**

### 4.2 Topography Characterization

The nanoscale imaging of the wet-etched DVD substrate and the metasurfaces were carried out via SEM (FEI Quanta 200 FEG), FIB-SEM (FEI Nova NanoLab 600 DualBeam), and AFM (Asylum, Oxford Instrument, U.K.). Prior to imaging, the surfaces were cleaned with ethanol and water, respectively, and dried with gentle air. Detailed topography characterization parameters were provided in the Supporting Information **.**

### 4.3 Simulations

#### 4.3.1 Numerical Flow Simulations

The effect of shear stress on protein adhesion kinetics in the microfluidic chip was simulated using COMSOL Multiphysics 5.6. software according to Navier–Stokes equations by considering channel height, flow rate, and adhesion time.^62^ Detailed descriptions of the simulation design and outcomes were discussed in the **Supporting Information**.

#### 4.3.2 FDTD Simulations

The electromagnetic response of the plasmonic metasurface was studied using commercial FDTD software. The two-dimensional (2D) structural profile used in the simulation was directly imported from the AFM topography of the metasurface. The detailed simulation parameters are provided in the **Supporting Information**.

#### 4.3.3 FDTD Modeling of the Dipole

The contribution of avidin-FITC to the absorption spectra of the metasurface was modeled by placing an electric dipole over the grating structure by using FDTD numerical software (Figure S12). The dipole emission was set to 516 nm to mimic the emission of avidin-FITC. The interdistance between the dipole and the metasurface was varied, and the resultant absorption spectra were observed.

### 4.4 Fabricating Microfluidic Chips

Microfluidic chip design consisted of three main stages: (i) poly­(methyl methacrylate) (PMMA, 2 mm thickness), (ii) double-sided adhesive film (DSA, 50 μm thickness), and (iii) the sensor. PMMA layer is composed of inlets and outlets for the introduction and removal of samples. DSA layer, on the other hand, is accommodated between the sensor and PMMA layer, hence providing the boundaries of microchannels. In this study, the DSA layer was used thoroughly. The PMMA layer was sonicated in a baker containing ethanol; rinsed with water; and then dried with compressed air. The PMMA and DSA layers were designed and cut by the CAD software (RDWorks) and a laser cutter device (LazerFix, Turkey).^63–65^

### 4.5 Surface Modifications

Considering the interdistance between the metasurface sensor and fluorescence emitters, three different surface modifications were selected: short, medium, and long. For short-distance modification, the metasurface was incubated overnight in a 3-MNHS (10 mM, ethanol) solution. Non-bound short-distance modifications were washed away with distilled water, and the metasurface was gently dried by using an air gun before microfluidic chip installation. Avidin (100 nM) was subsequently applied to the short-distance modification for approximately 50 min at a flow rate of 5 μL/min. Following avidin application, PBS was employed over the avidin-grafted short-distance modifications for 5 min to remove any nonbound avidin proteins. The same procedure was used to apply avidin-FITC (100 nM, PBS) to the short-distance modification.

For the medium- and long-distance modifications, SH-PEG 600-biotin and SH-PEG 2000-biotin were employed over the metasurface, respectively. In this manner, a 10 mM of either SH-PEG 600-biotin or SH-PEG 2000-biotin (distilled water) solution was introduced to the microfluidic channels for 20 min at a rate of 5 μL/min, followed by a 5 min wash with distilled water. Either avidin (100 nM) or fluorescently conjugated avidin (avidin-FITC and avidin-Texas Red) (100 nM, PBS), or streptavidin (streptavidin-QD 525 and streptavidin-QD 625) (100 nM, PBS) proteins were applied to the medium-distance modifications. These proteins were applied over the surface modifications using the previously explained avidin adhesion procedure. Similarly, a long-distance modification was also separately grafted with avidin and avidin-FITC using the same procedure.

### 4.6 XPS Analysis

We examined the formation of 3-MNHS, SH-PEG 600-biotin, and SH-PEG 2000-biotin layers on the sensor surface through an XPS analysis (K-Alpha XPS, Thermo Fisher Scientific, U.S.A). The experimental conditions, all the parameters, and data collection were acquired using the software (Thermo Advantage). In this study, C 1s, O 1s, N 1s, S 2p, and Au 4f scans were evaluated for all surface chemistry strategies.

### 4.7 Surface Plasmon Resonance Measurements

Spectroscopic measurements were performed with a portable optical setup and in-house software written on MATLAB GIU.

### 4.8 Fluorescence Microscopy Analysis

The adhesion of avidin-FITC was investigated by an upright fluorescence microscope (Zeiss AXIO equipped with an HBO100 laser source) for the medium-distance modification. To achieve this task, the microfluidic chip was placed under a fluorescence microscope and avidin-FITC was introduced on to the medium-distance modification. The following steps were visualized under flow conditions using the fluorescence microscope: (i) prior to avidin-FITC protein binding, (ii) after protein binding, and (iii) following the washing steps. On each step, the green fluorescence intensity of the entire image was taken into account for histogram calculations. After the washing steps, the avidin-FITC molecules were isolated. The analysis was performed by using NIH ImageJ software (USA). The avidin-FITC molecules were separately introduced onto a metasurface functionalized with each surface modification (short, medium, and long) by the following previously described steps. These isolated avidin-FITC molecules were captured for each surface modification, and a background subtraction (rolling ball radius of 50 pixels) was applied to every image. The fluorescence image of avidin-FITC molecules were cropped into a 4 × 4 pixel^2^ region of interests (ROIs) for intensity analysis. The normalized mean avidin-FITC intensities were obtained from the histogram analysis of five comparable ROIs for each surface modification.

## Supplementary Material



## Notes

Materials; topography imaging parameters (including FIB, SEM and AFM); fabrication of the plasmonic metasurface; morphological analysis of wet etched DVD; FDTD simulation of the metasurface; optical setup, numerical flow simulations; and XPS analysis of surface modifications (PDF)
