# Supplementary material for: Spatiotemporal Confinements of Distance-Dependent Emitters for Enhancing Plasmonic Signals
Source: ACS Appl Mater Interfaces. 2026 Feb 9;18(7):11774–84. doi: 10.1021/acsami.5c24749 (PMC12954659; doi:10.1021/acsami.5c24749)
Supplement: Supplementary file 1 [file am5c24749_si_001.pdf]

SUPPORTING INFORMATION

SPATIO-TEMPORAL CONFINEMENTS OF DISTANCE-DEPENDENT EMITTERS FOR ENHANCING PLASMONIC SIGNALS

Yusuf Aslan[1,2, §], Esma Derin[1,2, §], Kutay Sagdic[1,2], Timuçin Emre Tabaru[1, 2, 3], Ali Karatutlu [1,2,3], Bülend Ortaç [1,2], Fatih Inci [1,2,*]

[1] UNAM − National Nanotechnology Research Center, Bilkent University, 06800 Ankara, Turkey.

[2] Institute of Materials Science and Nanotechnology, Bilkent University, 06800 Ankara, Turkey

[3] Sivas University of Science and Technology, Mecnun Otyakmaz Street No:1 Sivas 58100, Turkey

[*] Corresponding author: finci@bilkent.edu.tr

[§] These authors contributed equally.

Table of Contents

1. Materials 3
2. Topography Imaging Parameters 3
3. Fabrication of the Plasmonic Metasurface 4
4. Morphological Analysis of Wet Etched DVD 5
5. FDTD Simulation of the Metasurface 6
6. Optical Setup 6
7. Numerical Flow Simulations 7
8. XPS Analysis of Surface Modifications 9
SUPPORTING FIGURES 12
SUPPORTING TABLES 24
Supplementary References 26

1. Materials

Methanol (99.7%), 3-mercaptopropanyl-N-hydroxy-succinamide ester (3-MNHS), Sigmatrix urine diluent (artificial urine), and bovine serum albumin (BSA) were purchased from Sigma-Aldrich (St. Louis, USA). Absolute ethanol (99.9%) and glycerol (85%) were obtained from Isolab Chemicals (Isolab, Istanbul, Türkiye). SH-PEG 600-biotin and SH-PEG 2000-biotin were purchased from Nanosoft Biotechnology LLC (NC, USA). Avidin protein was obtained from Thermo Scientific (Waltham, MA, USA). Avidin-FITC, avidin-Texas Red, streptavidin-QD 525, and streptavidin-QD 625 were purchased from Invitrogen (Carlsbad, CA, USA). Phosphate saline buffer (PBS) tablets were acquired from Biomatik (Ontario, Canada). The other organic solvents including ethanol (96%, Soltek), isopropanol alcohol (99.5%, Lobal Chemie), and acetone (99.5%, Birpa) were obtained from the companies stated above. All the chemicals were used without further purification. In addition, HP-brand (Palo Alto, CA, USA) digital versatile discs (DVDs) were utilized as a substrate for the fabrication of the plasmonic metasurfaces.

2. Topography Imaging Parameters

DVDs are composed of polycarbonate and they do not provide sufficient secondary electrons for SEM imaging. Therefore, clean DVD samples were coated with a 10 nm of gold-palladium (Au-Pd) using the Precision Etching and Coating System (PECS). During this procedure, the device parameters were adjusted to a coating density of 19.32 g/cm^3 and an acoustic resistance of 23.20 x 10^5 gm/cm^{2}s (acoustic impedance). Plasmonic metasurface was not coated with additional Au-Pd mixture. To further minimize electron

scattering and improve image stability, DVDs and plasmonic metasurfaces were mounted on SEM stubs using conductive carbon tapes.

FIB exposure can damage the groove structure of plasmonic metasurfaces even under imaging conditions. To prevent this, a 100 nm platinum layer was first deposited on the metasurface using electron-beam evaporation. An additional 1 μm platinum layer was then deposited to ensure a stable cross-sectional profile during the subsequent etching process. The platinum-coated surface was etched to a depth of approximately 2 μm using standard and cleaning cross-section modes sequentially. FIB-SEM images were then acquired in immersion mode.

For the AFM analysis, a cantilever (Nanosensors PPP-NCHR tip) with 42 N m^{-1} force constant was employed in AC Air Topography mode. Imaging was performed with a scan rate of 0.6 Hz, and were collected from the 2 μm x 2 μm area. The captured images were analyzed using Gwyddion software without any data manipulations including filters or smoothing.

3. Fabrication of the Plasmonic Metasurface

DVDs were employed as a base material of the metasurface due to their intrinsic nanoperiodic structures [1]. DVDs have a multi-layer structure, consisting of a top plastic surface, metal-coating, photoresist layers, and nanoperiodic surface grating made of polycarbonate-plastic layer [2]. The coating layers (undesired metal and photoresist layers) on DVDs were removed by physical and chemical processes [2]. Briefly, (1) the top layer was taken off using a knife; (2) the remaining metal-coating was removed by air pressure; (3) the photoresist layer was eliminated using a mixture of ethanol (EtOH) and methanol

(MetOH) (1:1:, v:v), followed by a washing step with distilled water; and (4) finally, the surface was chemically etched with a mixture of acetone and isopropanol (1:4; v:v) for 60 seconds to arrange the height and width of nanoperiodic structures. Then, the processed DVD was respectively coated with titanium (10 nm), silver (30 nm), and gold (15 nm) through thermal evaporator (MiDAS PVD 3T, VAKSIS, Ankara, Turkey) under 1x10-6 Torr of pressure. Tungsten boats (Midwest Tungsten Service) (Willowbrook, IL, USA) were preferred for metal sources. Titanium layer was coated at approximately 1.5 Å/s to prevent to prolonged heat exposure to polycarbonate substrate. Silver and gold layers were initially coated with 0.1 Å/s for the first 5 nm and the evaporation rate was gradually elevated approximately 1 Å/s. Once the metasurface was developed, the plasmonic metasurface surface was cut into 1.5 cm x 1.5 cm for further experiments.

4. Morphological Analysis of Wet Etched DVD

As reported in the literature [2], polycarbonate material of the DVD was chemically etched before the addition of plasmonic metals. For this, a 1:4 ratio of acetone-isopropanol mixture was applied to the cleaned disk surfaces with varying durations (10, 20, 30, 60, 90, and 120 seconds) (**Figure S1a-f**). It can be seen from past studies [2] that the etching time affects the width and space of the nano-grating structures. As the etching time increases, the width decreases, whereas the space between the gratings increases. As an example, over the course of 10 seconds of chemical etching the width of the grating was 750 nm, and the height was 160 nm (**Figure S1g**). When extending the duration up to 20 seconds, the grating width decreased to 530 nm, and the space in the structure expanded (**Figure S1h**). However, there were no significant changes in the height. When

the etching time was increased to 30, 60, and 90 seconds, the period of the gratings was observed as 740 nm, and the height was 160 nm on average (**Figure S1i-l**).

5. FDTD Simulation of the Metasurface

The simulation included a periodic plane wave with 300-1000 nm wavelength range. The plane wave was set to p-polarization. Mesh size was chosen 0.5 nm in Y direction and 2 nm in X direction. Boundary conditions were set to periodic for X direction and perfectly matched layer (PML) for Y direction. The bare substrate was simulated as a dielectric modelling the constant refractive index of polycarbonate (n=1.58). Over the polycarbonate substrate, titanium (10 nm), silver (30 nm), and gold (15 nm) layers were substituted. The dispersion properties of the metal layers were simulated using Palik models [3]. The refractive index of the medium was selected as 1.33 to model aqueous solutions.

6. Optical Setup

The optical setup was designed to collect and monitor the reflected plasmonic resonance shift from the metasurface (**Figure S4**). A broadband light source coupled to an optical fiber (Thorlabs OSL2) was used to excite surface plasmons on the metasurface. The light was collimated and linearly polarized (510-800 nm) before being directed by a 50:50 beam splitter onto the surface. The reflected light was then collected by a portable spectrometer (Compact CCD Spectrometer, CCS175, Thorlabs) after passing back through the lens, beam splitter, and a cosine corrector. The collected spectra were analyzed using a custom-designed MATLAB GUI, and the plasmonic resonance was tracked through continuous (5th degree) polynomial fitting.

In all measurements, the setup scanned a broad wavelength range from 500 to 1100 nm (<0.6 nm FWHM @ 633 nm). Reflected light was converted to absorbance using A=1-R-T, where transmittance was negligible (T≈0). For spectral analysis of the plasmonic response, a blank channel (metasurface–air–PMMA) was first measured and recorded as a reference data. After the introduction of measurement buffer (such as PBS or artificial urine) and the sample (such as avidin), the corresponding spectrum was recorded. The net spectral shift caused by the refractive index of the sample over the metasurface was then obtained by subtracting the blank data from the sample data.

7. Numerical Flow Simulations

The microfluidic design has a 4.5/10.2 aspect ratio (4.5 mm x 10.2 mm x 50 μm, Width x Length x Height) and 0.65 mm diameter inlet and outlet (**Figure S7a**). Theoretically, the shear stress profile is higher at the edges of the microfluidic chip [4], hence we have correspondingly numbered local regions (12 edges and corners) to analyze shear stress inside the chip through the red arrows. As a note, t = 0 s states that the moment occurs when the flow enters the chip from the inlet and then exits from the outlet for the first time. Initially, the shear stress was evaluated by employing different flow rates (5, 10, 20, 50, and 100 μL min^{-1}) while keeping the channel height (50 μm) constant (**Figure S7b**). The magnitude of shear stress has increased at the edges of the channel, whilst it decreased at the corners. Additionally, the shape of the shear stress profile from the inlet (1) to the outlet (7) was similar to the opposite side of the channel (the region numbered (8) to (12)), so that we have split symmetrical profiles with a red dashed line to make analysis easier. The lowest shear stress profile was observed at 5 μL min^{-1} of flow rate, achieving shear stress of ≤ 0.18 dyne cm^{-2}, and the shear stress increased ~2-21

times more than that of the lowest flow rate. The shear stress values were ≤ 0.37, ≤ 0.72, ≤ 1.81, and ≤ 3.71 dyne cm^{-2} for 10, 20, 50, and 100 μL min^{-1} flow rates, respectively. When the profile regions were examined at 5 μL min^{-1} particularly, the shear stress value was 0.01 dyne cm^{-2} in the entrance region of the chip (1), 0.18 dyne cm^{-2} in the place of (2), 0.01 dyne cm^{-2} in the corner numbered (3). 0.06 dyne cm^{-2} on the side of (4), 0.02 dyne cm^{-2} on the corner of (5), 0.09 dyne cm^{-2} in the region of (6), and 0.01 dyne cm^{-2} in the chip exit region (7).

As per the results, we determined to continue with a 5 μL min^{-1} of flow rate for further simulations due to the low shear stress profile that enables more efficient immobilization and quantitative analysis. The simulation was hence repeated at 5 μL min^{-1} for different channel heights (25, 50, 100 μm) for assessing the effect of height onto surface shear stress while keeping the time as t = 0 s (**Figure S7c**). The highest shear stress profile was observed at 25 μm of height, reaching the shear stress of ≤ 0.37 dyne cm^{-2}, and the stress values were observed as ≤ 0.18, and ≤ 0.09 dyne cm^{-2} for 50 and 100 μL min^{-1} of flow rates, respectively. Typical shear stress profiles of microfluidic devices along with 50 and 100 μm of channel heights were more suitable for biomolecule immobilization due to low shear stress profiles. Considering the usage of a lower volume material and the higher interactions of surface chemistry-biomolecule, the optimum parameter for the channel height was determined as 50 μm.

Afterwards, the shear stress profile was examined throughout the experimental flow duration (0-1800 s) while keeping the flow rate (5 μL min^{-1}) and the channel height (50 μm) constant (**Figure S7d**). In this manner, the overall shear stress profile was stabilized, and the magnitude of surface stress decreased after the initial condition (t = 0 s). Then,

the surface shear stress profile (t = 0 s) was depicted in **Figure S7e**. The middle region of the channel had a lower shear stress intensity, whilst the inlet and outlet regions had a higher shear stress profile with 0.47 dyne cm^{-2} maximum, and 2.3 x 10^{-5} minimum values. After accomplishing the simulations, the parameters were defined as 50 μm of microchannel height under the flow rate of 5 μL min^{-1}, hence continuing all the experiments with these parameters.

8. XPS Analysis of Surface Modifications

Here, we evaluated the composition of surface modifications applied onto the sensor surface in terms of C1s, O1s, N1s, S2p, and Au4f scans (**Figure S8**). Considering the case of 3-MNHS in the C1s scan, the sensor provided three peaks accounting for C=O at 288.36 eV, C-O at 284.73 eV, and C-C/C-H at 284.73 eV, which are the major chemical groups in the molecular structure of 3-MNHS (**Figure S8a**). In the O1s scan, two peaks were detected, and they belonged to C=O at 534.21 eV and C-O at 531.71 eV (**Figure S8b**). The chemical structure of the 3-MNHS contains nitrogen in the succinimide side; therefore, the N1s region is analyzed. As a result, imide and amide groups were identified with peaks at 401.25 eV and 399.29 eV, respectively **(Figure S8c)**. 3-MNHS also contains a thiol group, which is attached to the gold surface, forming a metal dative bond. The related binding energies of the S2p region were stated as 162.88 eV (Au-S) and 161.66 eV (S-C) **(Figure S8d)**. Regarding the Au4f, the gold surface presented two peaks associated with Au ($4f_{5/2}$) and Au ($4f_{7/2}$) at the binding energies of 87.27 and 83.60 eV, respectively (**Figure S8e**). Overall, the C1s provided a higher atomic ratio due to the carbon backbone in the 3-MNHS structure, and the O1s were the second highest owing to C=O groups at the carbon backbone and the succinimide end (**Figure S8f**).

In **Figure S8g-l**, the same elements were scanned for the medium-distance modification, which was SH-PEG 600-biotin. Considering SH-PEG 600-biotin in the C1s scan, we observed three peaks associated with C=O at 288.08 eV, C-O at 285.79 eV, and C-C/C-H at 284.38 eV (**Figure S8g**). Comparing these energies with the ones observed in the case of 3-MNHS, the peak intensity of the C=O bond was lower. On the other hand, the intensity of the C-O bond was higher than that of 3-MNHS, and the resulting binding energy was shifted ~1 eV. In addition, the O1s scan for this polymer-modified surface resulted in two peaks associated with C=O at 533.08 eV and C-O at 531.84 eV (**Figure S8h**). Again, comparing them with the ones in the case of 3-MNHS, the binding energy of the C=O bond was shifted ~1.1 eV. Since these polymers contain biotin proteins, the N1s scan with the energy of 400 eV could be stemmed from peptide bond (–NH–C(=O)–). However, the N1s peak was negligible for SH-PEGs as reported in the literature [5] due to a lack of thickness and low surface coverage (**Figure S8i**). Due to the thiol-ends of SH-PEG 600-biotin molecules, the S2p scan provided two peaks for Au-S at 161.75 eV and for S-C at 160.12 eV; yet they were shallow peaks with lower intensities compared to the data derived from the case of 3-MNHS. Regarding the Au4f, the sensor presented two peaks associated with Au ($4f_{5/2}$) and Au ($4f_{7/2}$) at the binding energies of 86.74 and 83.07 eV, respectively (**Figure S8k**). Their binding energies were shifted ~0.5 eV compared to the case of 3-MNHS.

Assessing the SH-PEG 2000-biotin on the course of the C1s scan, three peaks associated with C=O, C-O, and C-C/C-H were observed at the binding energies of 287.30, 285.25, and 283.86 eV, respectively (**Figure S8m**). In addition, the O1s scan for this surface resulted in two peaks associated with C=O at 533.49 eV and C-O at 531.41 eV

(**Figure S8n**). Likewise, we did not observe any signals for SH-PEG 2000-biotin on the course of the N1s scan [5] (**Figure S8o**). Owing to the thiol-end, the S2p scan provided two peaks for Au-S at 162.13 eV and for S-C at 160.80 eV. Similarly, they were shallow peaks with lower intensities than those of 3-MNHS data (**Figure S8p**). Moreover, the binding energies of these bonds were shifted ~0.6 eV in comparison to the data collected from the SH-PEG 600-biotin-modified sensor. Regarding the scan of Au4f, the sensor exhibited two peaks associated with Au ($4f_{5/2}$) and Au ($4f_{7/2}$) at the binding energies of 86.61 and 82.93 eV, respectively (**Figure S8r**), which had similar binding energies with the other polymer-decorated surface. In contrast to C1s, O1s, N1s, and S2p, the intensities of the Au4f peaks were lower since the usual information depth of XPS is limited to 1-10 nm [6], where a gold layer is possibly hidden by the surface chemistry. Considering both polymeric molecules, the C1s stated more atomic ratio due to the carbon backbone of the molecules, and the O1s was the second highest since the molecule has O-H and C=O groups (**Figures S8l and S8s**).

SUPPORTING FIGURES

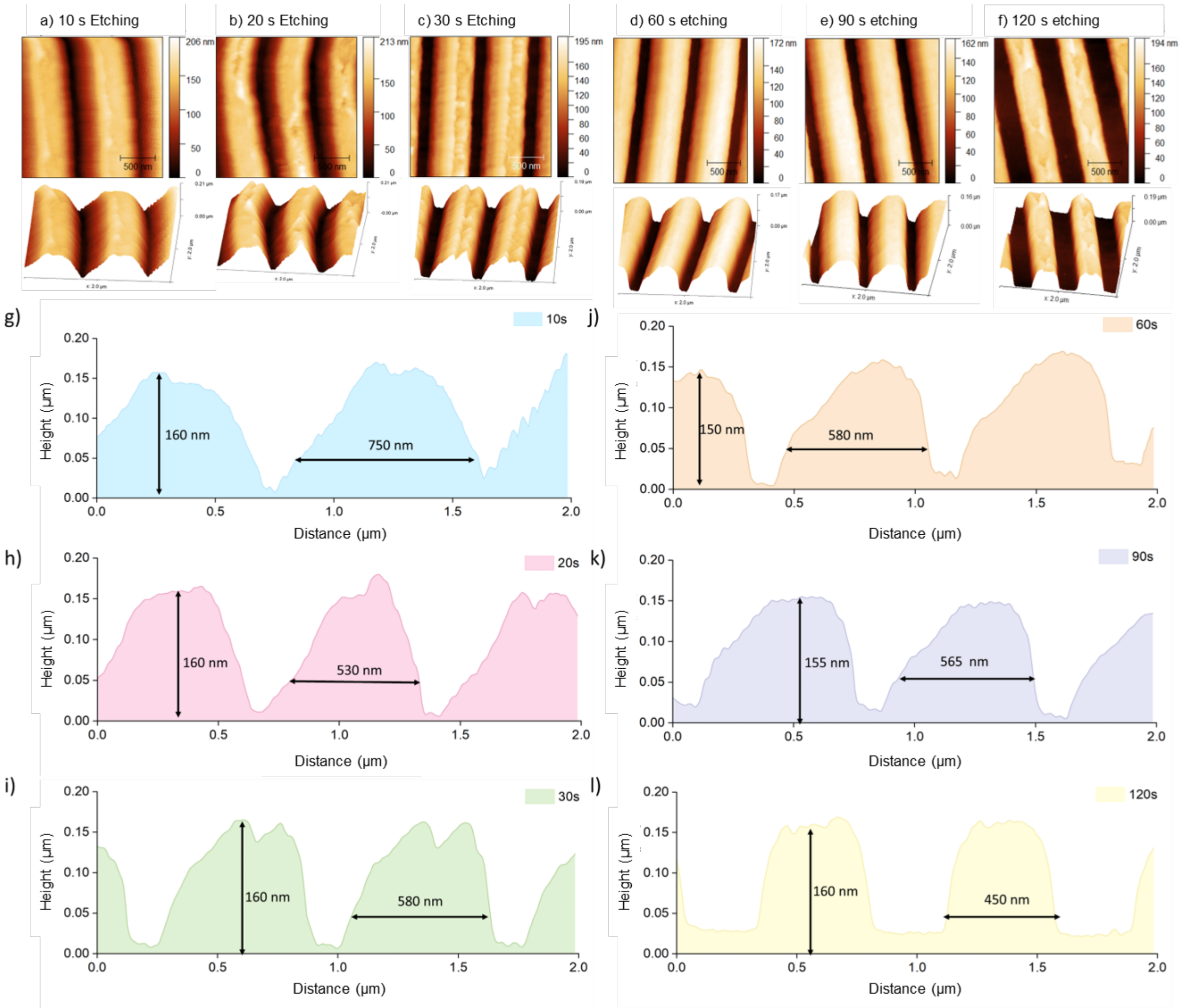

Figure S1: The morphological effects of chemical etching on bare DVDs were investigated using AFM. Two-dimensional (top) and three-dimensional (bottom) AFM images show the changes in the grating structure after varying etching times: (a-f) 10-120 seconds. The line profiles illustrate the changes in the height and width of the grating structures after varying etching times: (g-l) 10-120 seconds.

a)

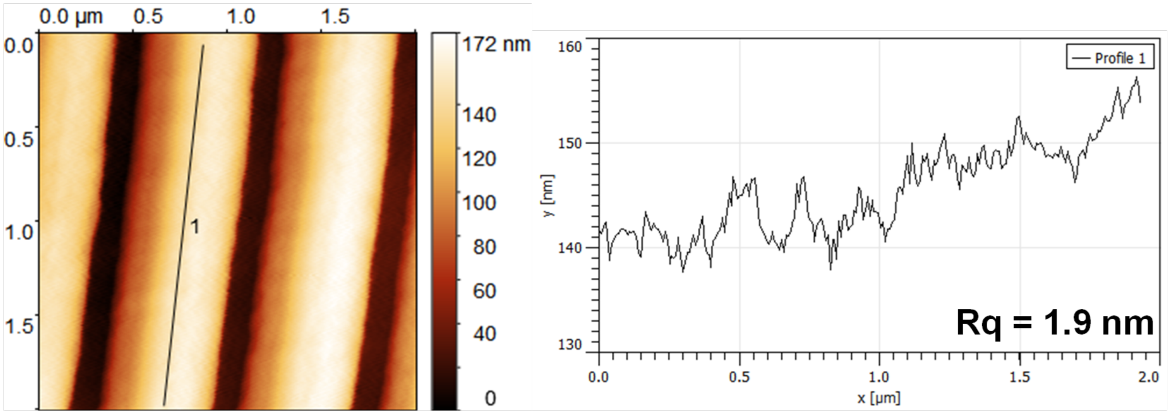

b)

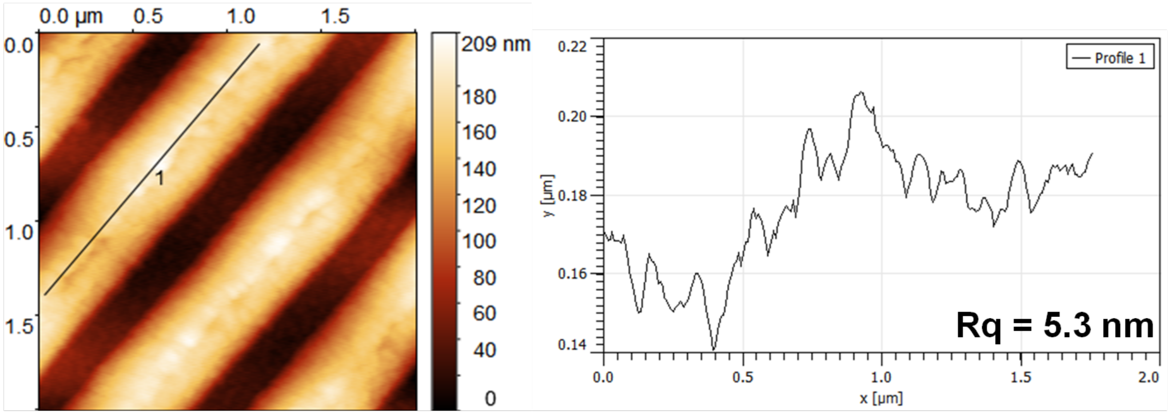

Figure S2: Surface roughness analysis of the DVD substrate and plasmonic metasurface. AFM images (left) show the nanoscale grating structure, and the extracted line profiles (right) reveal the corresponding root-mean-square (RMS) roughness values (Rq).

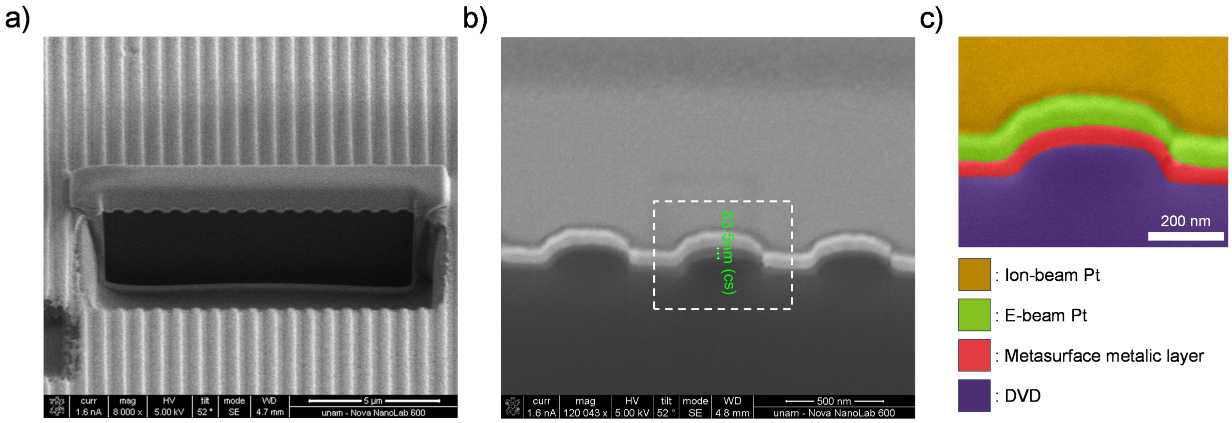

Figure S3: FIB cross-sectional analysis of the metasurface. (a) SEM image showing ion- and electron-beam deposited platinum protection layers. (b) Cross-section revealing a total metallic layer thickness of ~63.5 nm over the metasurface. (c) Pseudo-colored SEM image highlighting the distinct layers within the cross-section.

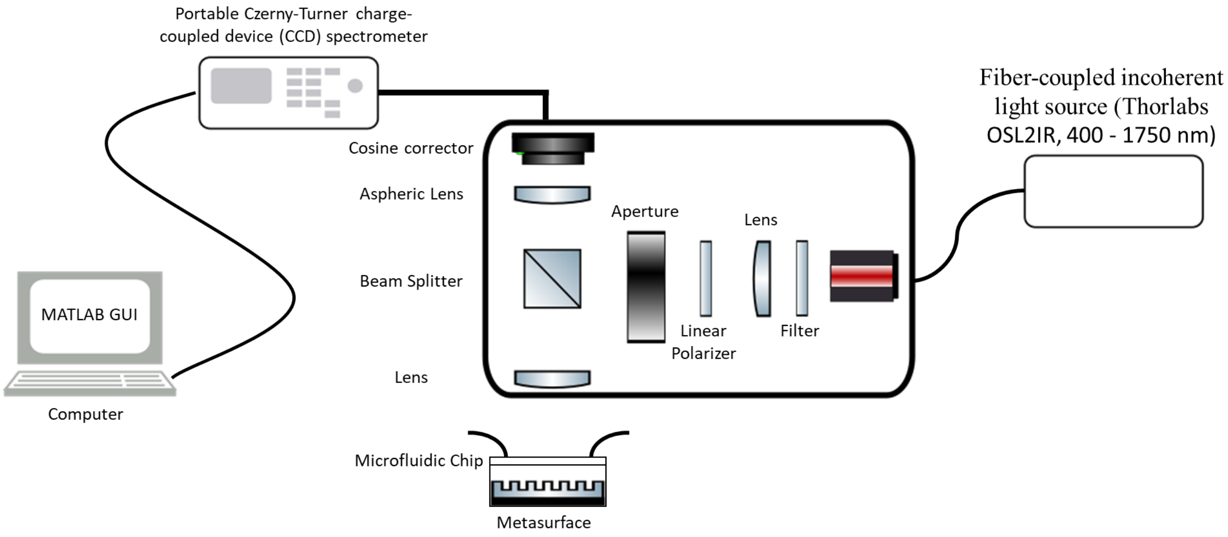

Figure S4: Schematic illustration of the optical set-up used for monitoring wavelength shift.

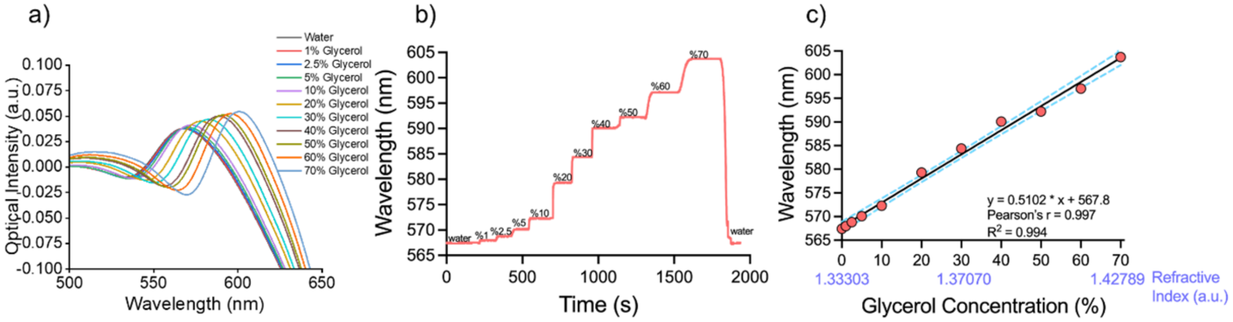

Figure S5: Glycerol solution experiments. The changes in resonance wavelength according to different glycerol solutions (1-70%) are depicted by analyzing a) end measurements and b) real-time data. (c) Regression analysis and linearity of resonance wavelength shifts according to refractive index of glycerol solutions.

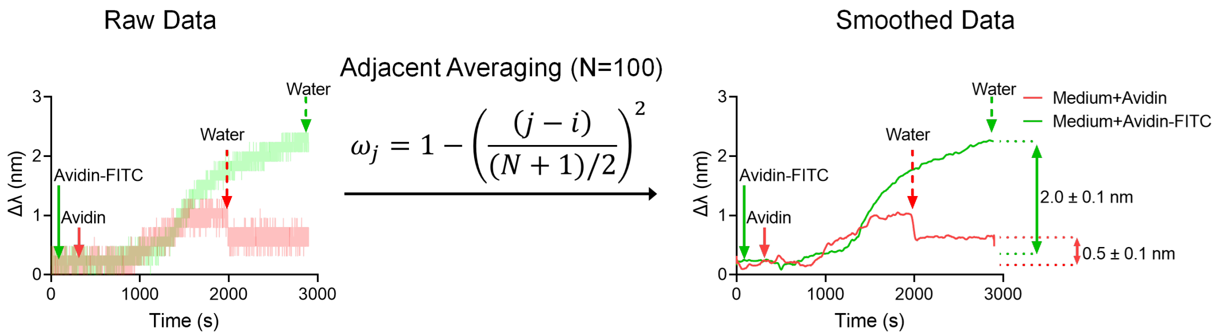

Figure S6: Adjacent averaging of the plasmonic resonance shift converts raw spectral data to smoothed data.

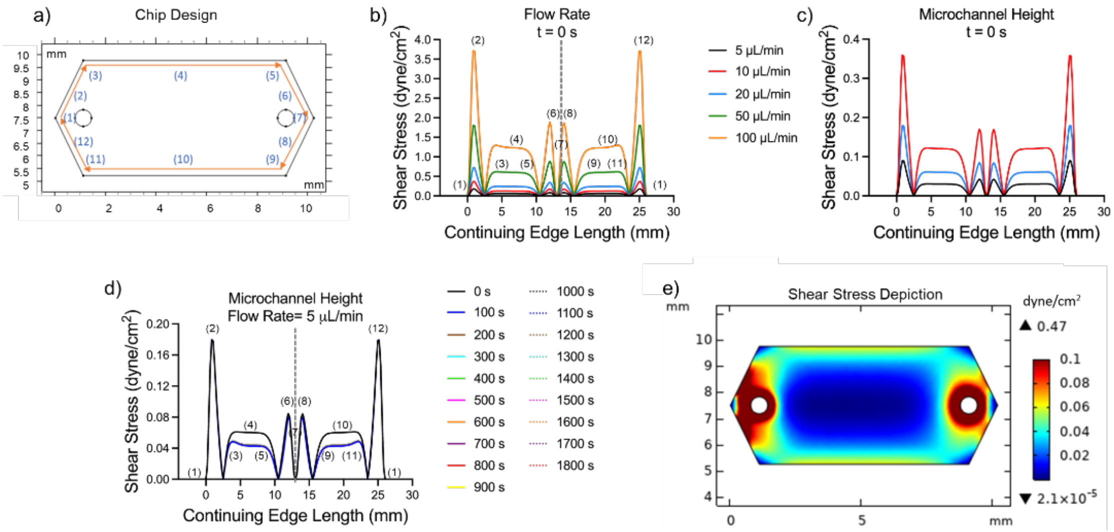

Figure S7: Numerical flow simulations within the microchannel are presented. a) A schematic representation of the microfluidic channel design with arrow indicators for shear stress analysis. b) Velocity profiles according to the arrow indicators are shown for the flow rates of 5, 10, 20, 50, and 100 μL/min at t=0 s. c) Shear stress profiles according to the arrow indicators are shown for the channel heights of 25, 50, and 100 μm under the flow rate of 5 μL/min at t=0 s. d) The same shear stress profile measured over a period from 0 to 1800 s. e) A heatmap illustrating the surface shear stress profile at t=0 s, with a flow rate of 5 μL/min applied to the microfluidic chip with a 50 μm channel height.

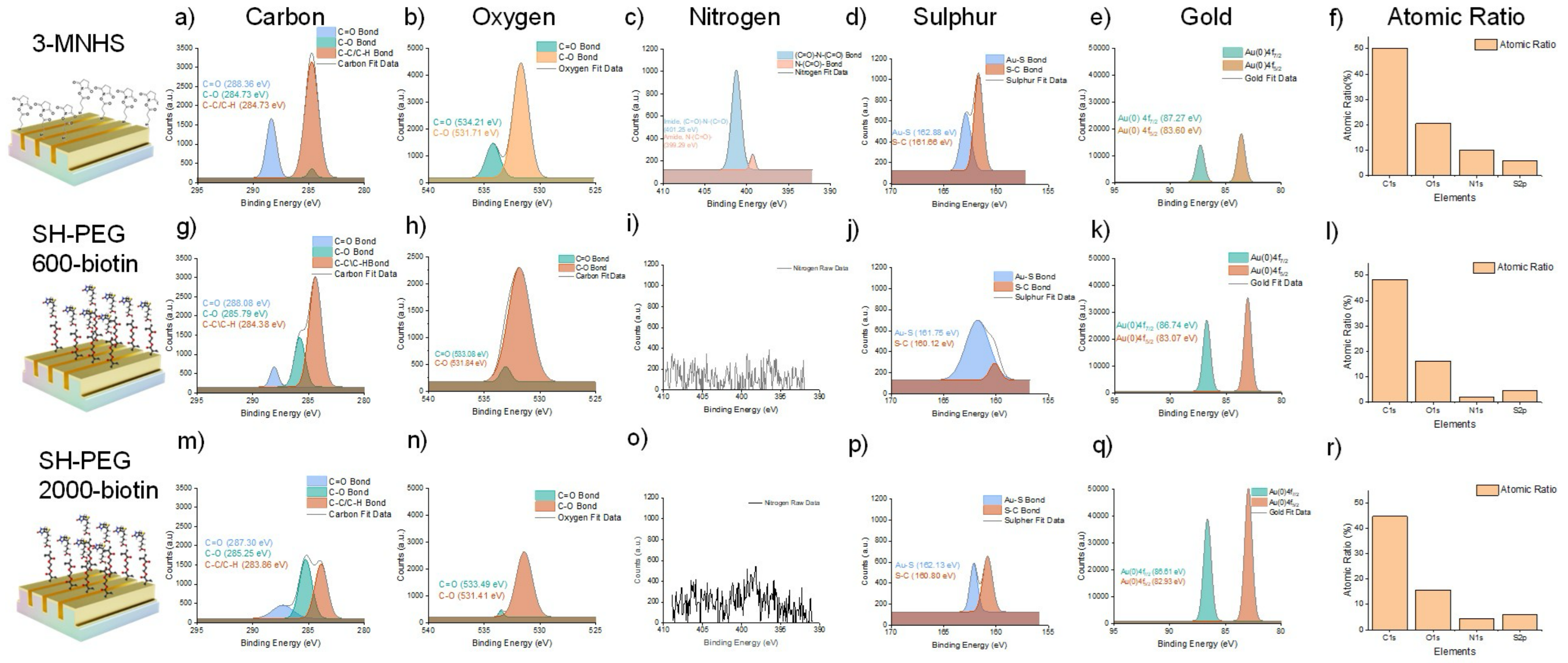

Figure S8: XPS characterization of surface modifications on the metasurface is demonstrated. The chemical structure of the short-distance modification (3-MNHS) is evaluated with XPS spectra in the (a) C1s, (b) O1s, (c) N1s, (d) S2p, and (e) Au4f regions. The resultant (f) atomic ratio for the short-distance modification is demonstrated. The chemical structure of the medium-distance modification (SH-PEG 600-Biotin) is evaluated in the (g) C1s, (h) O1s, (i) N1s, (j) S2p, and (k) Au4f regions. The chemical structure of the long-distance modification (SH-PEG 2000-Biotin) is evaluated in (m) C1s, (n) O1s, (o) N1s, (p) S2p, and (q) Au4f regions. The resultant (r) atomic ratio for the long-distance modification is demonstrated.

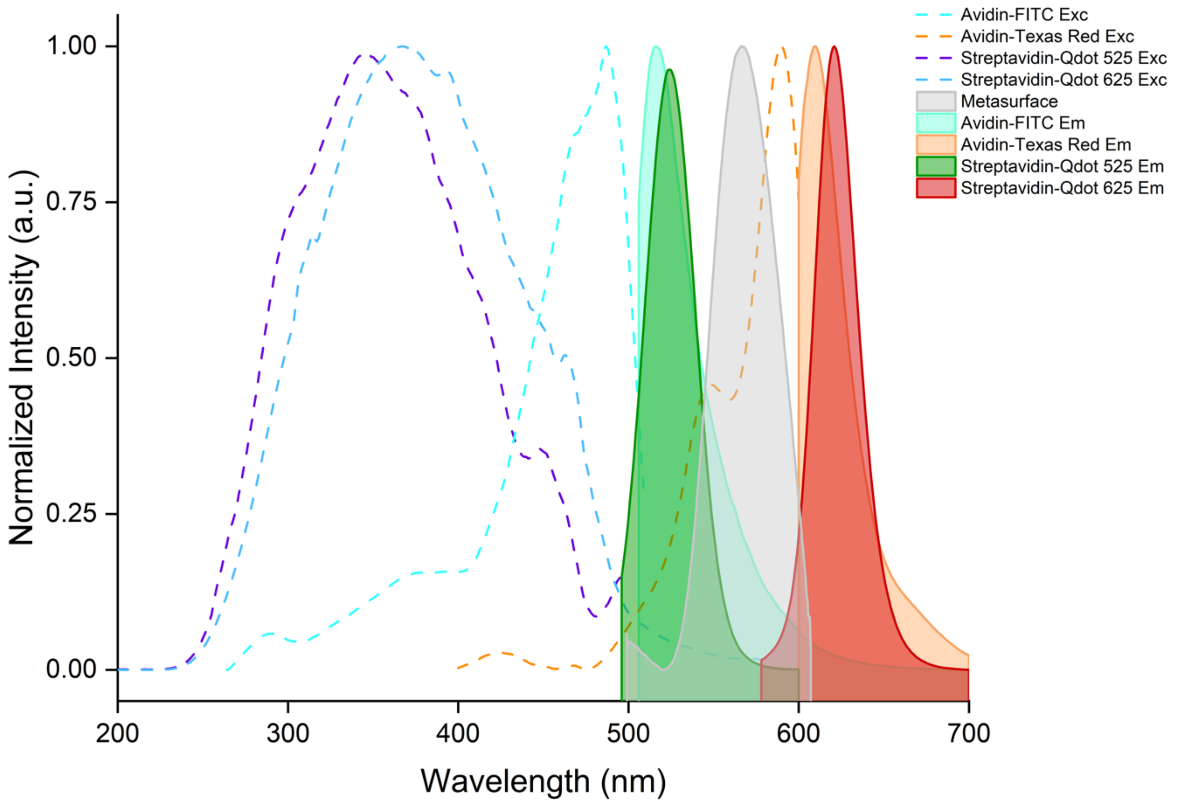

Figure S9: Spectral overlap between the fluorescence emitters (avidin-FITC, avidin-Texas Red, streptavidin-QD 525, and streptavidin-QD 625) and plasmonic metasurface. The excitation and emission spectrum of the fluorescence emitters were acquired by time-resolved fluorescence (TRF).

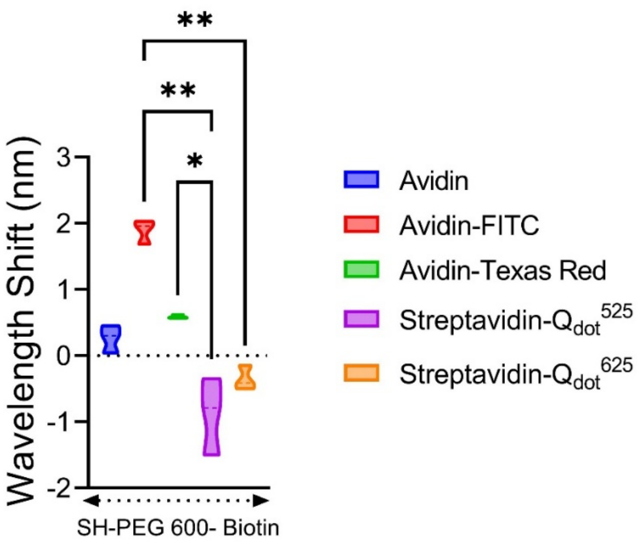

Figure S10: Violin-box graph is demonstrated for each emitter on medium-distance modification. The data is also evaluated by performing a non-parametric Kruskal-Wallis statistical analysis, and the statistical differences are shown with an asterisk (n=3, *p<0.05, ** p<0.01).

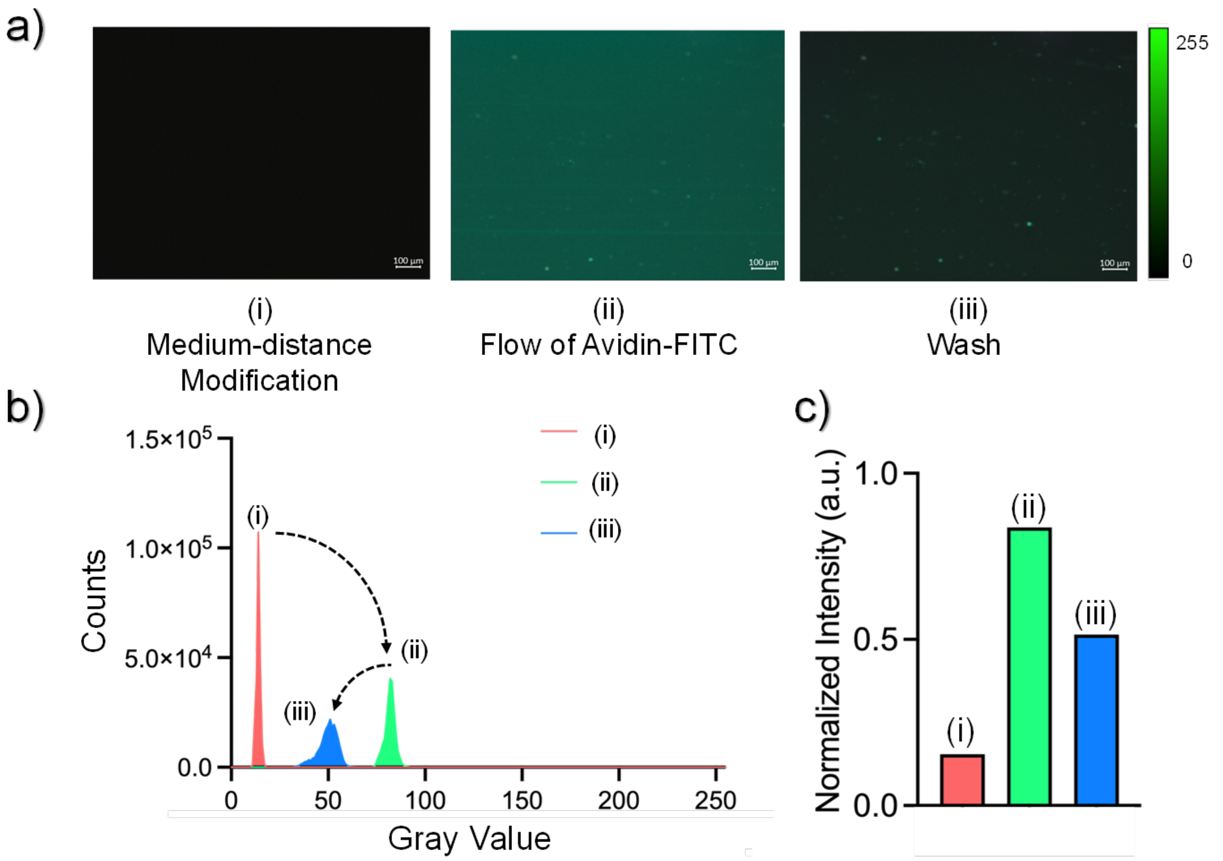

Figure S11: Fluorescence microscopy analysis of avidin-FITC immobilization on the medium-distance modified metasurfaces. (a) The fluorescence images under three conditions: (i) the medium-distance modification; (ii) after the introduction of avidin-FITC, and (iii) after PBS wash. (b) Corresponding fluorescence intensity histograms for each condition, and (c) normalized mean gray intensity for each condition.

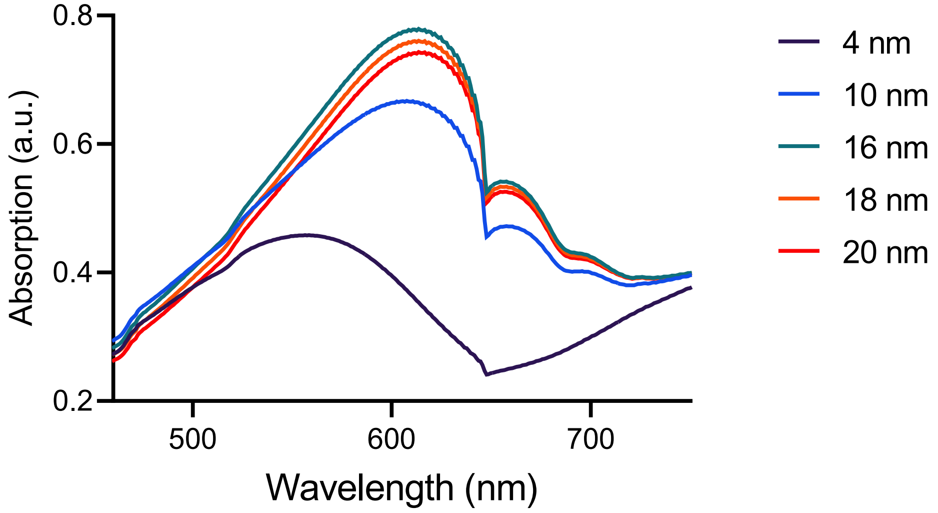

Figure S12: FDTD simulated absorption spectrum of dipole (λ=516 nm) above the metasurface structure with varied interdistance.

SUPPORTING TABLES

Table S1: Investigating FOM values of unmodified sensors while applying various concentrations of glycerol.

Glycerol Concentrations (%)	Refractive Index (RIU)	FWHM (nm)	FOM (1/RIU)
0	1.333	30.54	12.54912
1	1.334	30.28	12.65687
2.5	1.336	29.14	13.15202
5	1.339	28.58	13.40973
10	1.345	27.97	13.70218
20	1.358	27.32	14.02818
30	1.371	26.94	14.22606
40	1.384	26.01	14.73472
50	1.398	25.45	15.05894
60	1.413	24.9	15.39157
70	1.428	23.92	16.02216

Table S2: Investigation of FOM values of modified surface (SH-PEG 600-biotin+avidin-FITC immobilized surface)

Glycerol Concentration (%)	Refractive Index (RIU)	FWHM (nm)	FOM (1/RIU)
0	1.333	20.76071	16.30358
1	1.334	20.65546	16.38666
2.5	1.336	20.63678	16.40149
5	1.339	20.02716	16.90075
10	1.345	19.51936	17.34042
20	1.358	18.48498	18.31076
30	1.371	18.13052	18.66874
40	1.384	17.55106	19.2851
50	1.398	16.67466	20.2987
60	1.413	16.5729	20.42334
70	1.428	16.2797	20.79117

Supplementary References

(1) Mataji-Kojouri, A.; Ozen, M. O.; Shahabadi, M.; Inci, F.; Demirci, U. Entangled Nanoplasmonic Cavities for Estimating Thickness of Surface-Adsorbed Layers. *ACS Nano* **2020**. https://doi.org/10.1021/acsnano.0c02797.

(2) Ahmed, R.; Ozen, M. O.; Karaaslan, M. G.; Prator, C. A.; Thanh, C.; Kumar, S.; Torres, L.; Iyer, N.; Munter, S.; Southern, S.; Henrich, T. J.; Inci, F.; Demirci, U. Tunable Fano-Resonant Metasurfaces on a Disposable Plastic-Template for Multimodal and Multiplex Biosensing. *Adv. Mater.* **2020**. https://doi.org/10.1002/adma.201907160.

(3) Handbook of Optical Constants of Solids I-III. **1998**.

(4) Kim, T. H.; Lee, J. M.; Ahrberg, C. D.; Chung, B. G. Development of the Microfluidic Device to Regulate Shear Stress Gradients. *BioChip J. 2018 124* **2018**, *12* (4), 294–303. https://doi.org/10.1007/S13206-018-2407-9.

(5) Ihalainen, P.; Majumdar, H.; Viitala, T.; Törngren, B.; Närjeoja, T.; Määttänen, A.; Sarfraz, J.; Härmä, H.; Yliperttula, M.; Österbacka, R.; Peltonen, J. Application of Paper-Supported Printed Gold Electrodes for Impedimetric Immunosensor Development. *Biosensors* **2013**, *3* (1), 1–17. https://doi.org/10.3390/bios3010001.

(6) Gilbert, J. B.; Rubner, M. F.; Cohen, R. E. Depth-Profiling X-Ray Photoelectron Spectroscopy (XPS) Analysis of Interlayer Diffusion in Polyelectrolyte Multilayers. *Proc. Natl. Acad. Sci. U. S. A.* **2013**, *110* (17), 6651–6656. https://doi.org/10.1073/pnas.1222325110.
